# Intracellular Information Processing through Encoding and Decoding of Dynamic Signaling Features

**DOI:** 10.1371/journal.pcbi.1004563

**Published:** 2015-10-22

**Authors:** Hirenkumar K. Makadia, James S. Schwaber, Rajanikanth Vadigepalli

**Affiliations:** Daniel Baugh Institute for Functional Genomics and Computational Biology, Department of Pathology, Anatomy and Cell Biology, Sidney Kimmel Medical College, Thomas Jefferson University, Philadelphia, Pennsylvania, United States of America; National Institutes of Health, UNITED STATES

## Abstract

Cell signaling dynamics and transcriptional regulatory activities are variable within specific cell types responding to an identical stimulus. In addition to studying the network interactions, there is much interest in utilizing single cell scale data to elucidate the non-random aspects of the variability involved in cellular decision making. Previous studies have considered the information transfer between the signaling and transcriptional domains based on an instantaneous relationship between the molecular activities. These studies predict a limited binary on/off encoding mechanism which underestimates the complexity of biological information processing, and hence the utility of single cell resolution data. Here we pursue a novel strategy that reformulates the information transfer problem as involving dynamic features of signaling rather than molecular abundances. We pursue a computational approach to test if and how the transcriptional regulatory activity patterns can be informative of the temporal history of signaling. Our analysis reveals (1) the dynamic features of signaling that significantly alter transcriptional regulatory patterns (encoding), and (2) the temporal history of signaling that can be inferred from single cell scale snapshots of transcriptional activity (decoding). Immediate early gene expression patterns were informative of signaling peak retention kinetics, whereas transcription factor activity patterns were informative of activation and deactivation kinetics of signaling. Moreover, the information processing aspects varied across the network, with each component encoding a selective subset of the dynamic signaling features. We developed novel sensitivity and information transfer maps to unravel the dynamic multiplexing of signaling features at each of these network components. Unsupervised clustering of the maps revealed two groups that aligned with network motifs distinguished by transcriptional feedforward vs feedback interactions. Our new computational methodology impacts the single cell scale experiments by identifying downstream snapshot measures required for inferring specific dynamical features of upstream signals involved in the regulation of cellular responses.

## Author Summary

Single cell studies have shown that differential patterns in the dynamics of signaling proteins, transcription factor activity, gene expression, etc. produce distinct downstream outcomes. The opposite also holds true where particular cellular outcomes have been found to be associated with the dynamical pattern of one or more signaling molecules. Signaling pathways, therefore, serve as signal processing units to inform specific downstream regulation. However, the functional capabilities of the dynamic aspects of signaling are not well understood. To address this issue, we developed a new approach that evaluates information processing between dynamic features in signaling patterns and transcriptional regulatory activity. Our work demonstrates that the information transfer occur through decoding of temporal history of signals rather than only through instantaneous correlations. Moreover, our results identify regulatory network motifs as the critical components in the information processing and filtering of variability in signaling dynamics to produce distinct patterns of downstream transcriptional responses. Our methodology can be broadly applied to single cell scale data on experimentally accessible downstream measures to infer dynamic aspects of upstream signaling.

## Introduction

Cells continuously sense a variety of physical and chemical signals and respond suitably to changes in their environment [1–3]. Depending on the nature of the environmental change, a subset of membrane receptors gets stimulated, eliciting specific signaling pathways and enabling cells to make informed decision downstream [4]. Recent studies indicate that the functional information to trigger a specific downstream response is typically carried through activity change in one or more signaling molecules, transcription factor activities or protein substrates [5–7]. Considering the multifunctionality of signaling pathways [8]), it has been argued that this mode of information transfer is more rich and complex than encoding it through merely binary on/off steady states [9–12]. For example, depending on the cell type, transient activation pattern of stimulated extracellular signal-regulated kinases (ERK) leads to cell proliferation, whereas sustained pattern of ERK may lead to cell differentiation [13]. However, the mechanism through which the functional information encoded within the temporal activity change is processed and decoded to very fine alterations in an upstream signaling pattern still remains unclear. Instantaneous activity change between upstream and downstream events has been typically considered to be the natural mechanism of information processing, thus, limiting our understanding of dynamical patterns [14–17]. Therefore, a challenging but relevant task is to elucidate the mechanism through which features or properties of signaling dynamics (time dependent activity-change in signals) encode information, and how cells decode this information via complex signaling pathways with high specificity, when variability is the norm.

Mounting evidence from single cell microscopy [18], flow cytometry [19], single cell PCR [20], single cell RNA-seq [21, 22], and single cell mass cytometry [23] studies reveals that variability in post-transcriptional or post-translational events, even within homogeneous condition, is ubiquitous [24–26]. Such results has generated considerable interest in employing single cell resolution data to elucidate the role of variability and characterize underlying mechanism of intracellular information processing [3, 27]. Since comprehensive network motif identification studies have unveiled a strong interdependence between dynamics, motif structure, and specific function [28], role of network topologies during information processing also needs to be considered [29]. For instance, feedforward loops have an ability to generate a transient activity or protect against brief fluctuations depending on the nature of their interactions [28], whereas positive feedback loops induces a response delay and hysteresis in the network dynamics [30]. Negative feedback mechanisms protect the information transfer by saturating the signaling pathways from upstream fluctuations [31]. In sum, precise orchestration of cellular events is a consequence of an efficient signaling modulation code involving upstream signaling dynamics and network motifs [1, 32, 33]. Hence, we hypothesize that functional information to direct cells towards specific responses is encoded in the temporal features of the signaling dynamics, and that the regulatory network motifs facilitate decoding this information.

**Fig 1:**
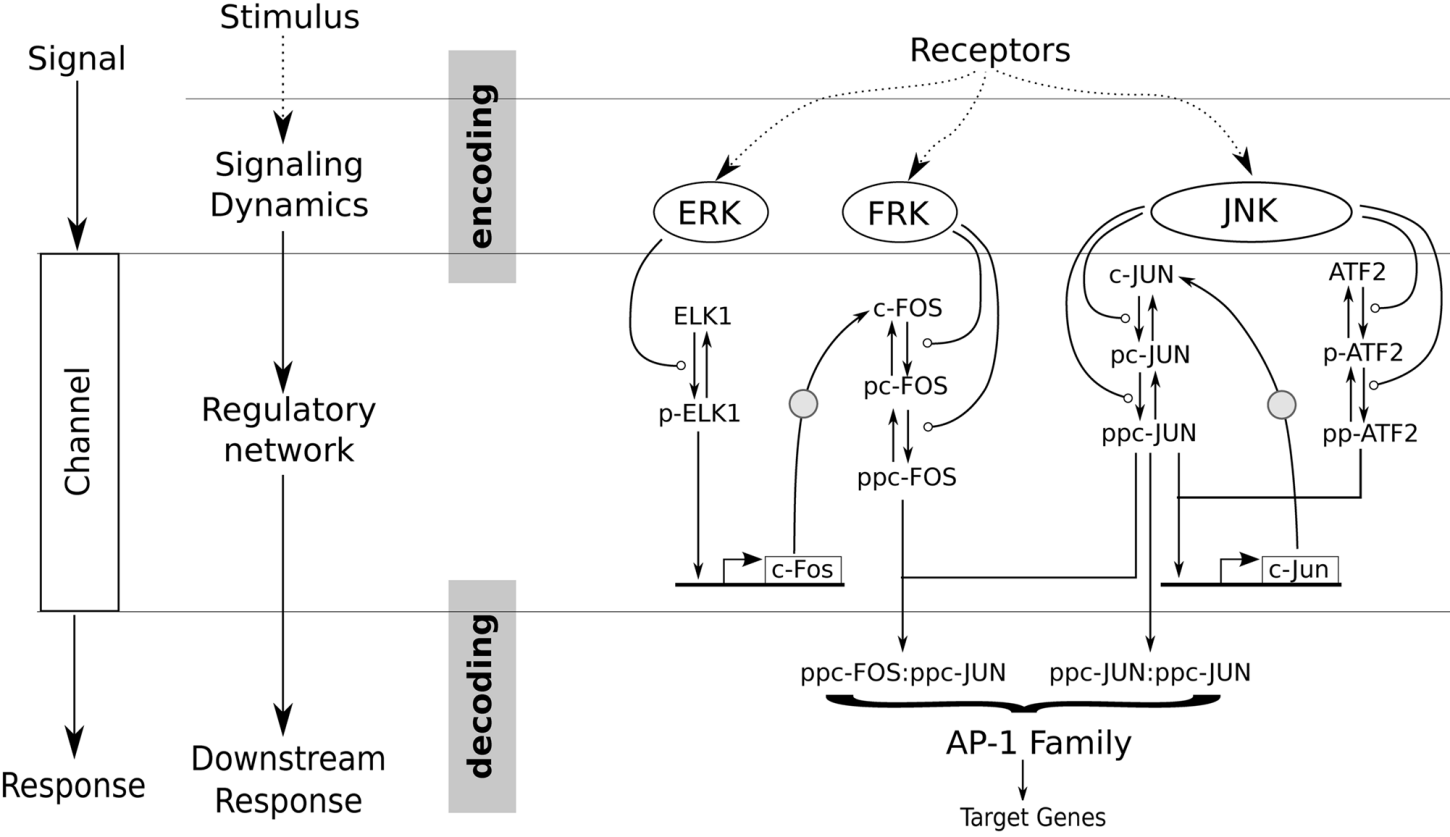
Integrated model of signaling dynamics and gene regulatory network. Comparison of the salient features of a modern communication network (left) to a biochemical signaling and gene regulatory network (right). The functional information in a receptor stimulus is encoded in temporal features of signals in a similar manner as in communication networks, where information is encoded in characteristic features (such as amplitude, phase, frequency, etc.) of the signals. These encoded signals are transmitted over the regulatory network (or channel) and decoded downstream (or receiver end).

We test our hypothesis through analysis of an integrated model of signaling dynamics and gene regulatory networks. Both gene regulatory networks and communication networks have a similar underlying operating principle [34, 35]. As shown in Fig 1 the goal is to encode relevant information in the characteristic features of the input signals and transmit them over a channel so they can be decoded at the receiving end [17, 36, 37]. Molecular signaling pathways elicited by receptor activation in cells which are involved in the regulation of physiological responses such as growth, differentiation and programmed apoptosis, among others [11, 38], have been shown to exhibit such properties [3, 9]. Unlike communication networks, there are limitations to the predictability of downstream responses in a biochemical network for any given input signal, and in particular at single cell levels [39]. This is because it is difficult to infer the strength of an input stimulus received by a single cell based on the instantaneous downstream responses alone.

**Fig 2:**
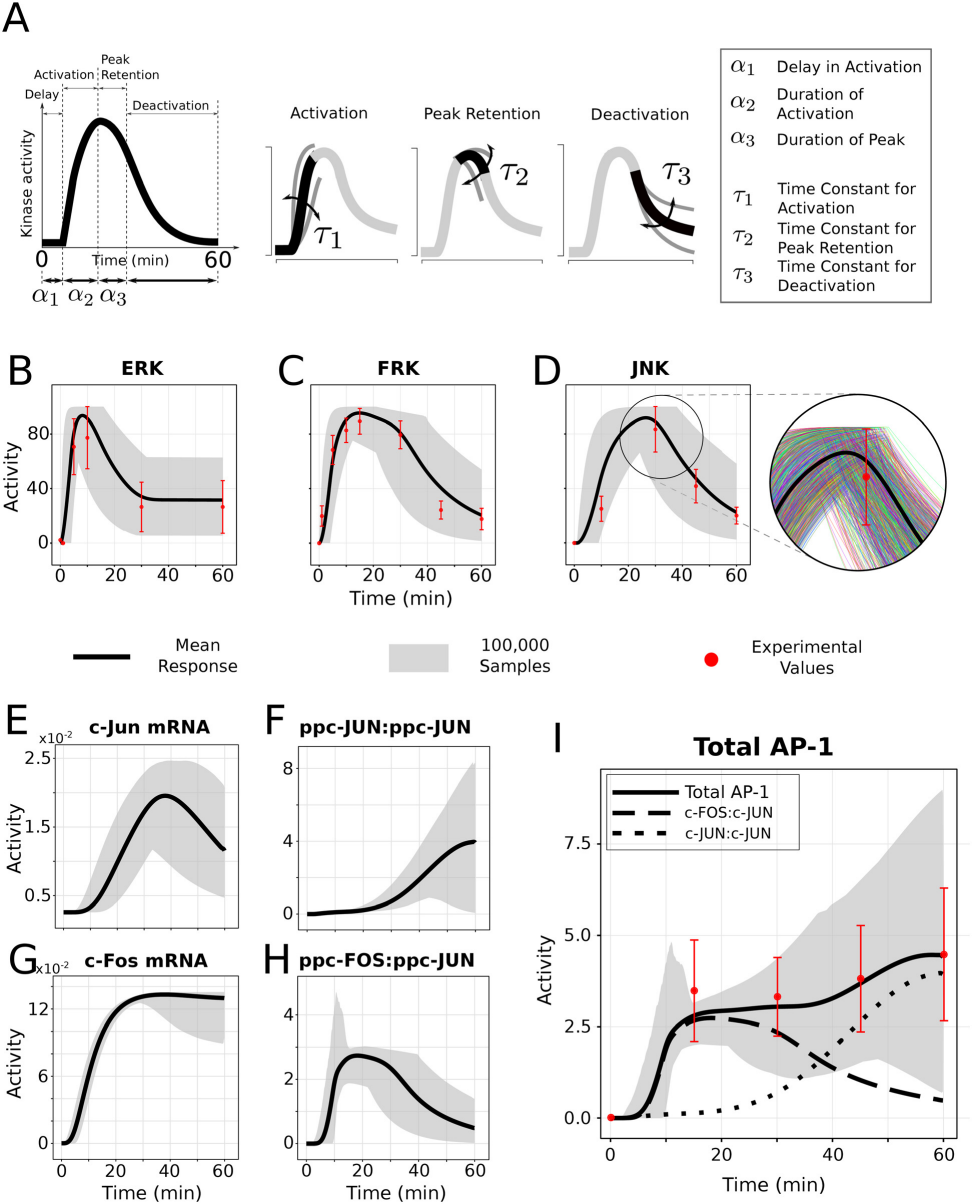
Functional information encoding through dynamical signaling features. (A) Illustration showing independent temporal features of dynamics of a transient signaling kinase in the phases of delay, activation, peak retention and deactivation kinetics. (See Methods and Table 1 for details) (B, C and D) Simultaneous variability in all six temporal features encompasses (gray region in the plot) the observed experimental variations in ERK, FRK and JNK activation (red error bars), respectively. The gray region is based on a total of 100,000 simultaneous variations in all eighteen signaling features, generated using a differential variation schema reported in Table 1. The red points and error bars in the plots show experimental data from neuronal cultures incubated with Angiotensin II (100 nM) to activate AT1R signaling for 0–60 min at 37°C. Time course data for ERK is taken from [93], whereas for FRK and JNK time course observations are from [94]. The extended figure on the right of the JNK plot illustrates a subset of 100,000 random profiles (colored lines) filling the gray region and bounded within the red error bars. (E,F,G, H and I) Simulations results from 0 to 60 minutes for each unique combination of three signaling profiles (100,000 random samples in total) for downstream responses of immediate early genes c-Jun and c-Fos, and transcription factors ppc-JUN:ppc-JUN, ppc-FOS:ppc-JUN and Total AP-1. Experimental data for Total AP-1 time course observations are taken from [46].

The goal of this manuscript is to assess how variation in dynamic signaling pattern influences downstream regulation at the single cell level. Identifying the mechanism of how a specific downstream phenotype could be triggered by different combinations of kinetic modulation in an upstream signaling pattern is critical to our understanding of the cellular decision making process. Here we employ a mechanistic ODE based model [40], that is comprised of multiple receptor stimulated signaling kinase cascades activating immediate early response genes (IEGs) (c-Fos and c-Jun), and transcription factors (TF) (AP-1), which turn on late response target genes (right side of Fig 1) [41]. This two-tier regulatory network is comprised of a feedback as well as a feedforward transcriptional network motif, making it a suitable candidate to test our hypothesis. We characterized a set of independent dynamical features for each input signal (ERK, FRK and JNK in Fig 1) through a novel phenomenological model (see Fig 2A for graphical illustration). Variance based global sensitivity analysis was used to study how uncertainty in signaling features affects uncertainty in downstream IEG and TF responses, and to also quantify the relative importance of each feature at every timepoint [40, 42]. Information theory was used to measure mutual information between signaling feature and the downstream gene regulatory responses. Mutual information quantifies the amount of information that is carried by a signaling feature, and allow us to evaluate its capacity to make a binary transcriptional decision downstream. We further use decision tree analysis [43], a predictive modeling technique, to identify decision rules for signaling features that significantly shape the IEG and TF response patterns. As single cell studies become more advanced, it has become more evident that cells may use dynamics of signaling species to encode important information that can be decoded to generate distinct cellular responses. We use our earlier published single cell gene expression data [20] for specific transcriptional target outputs to identify the upstream signaling features that are most likely to be transmitted into specific phenotypes via generation of distinct target gene expression levels.

Our results revealed that while signaling dynamics have many potential functional information encoding features, transcriptional regulatory network motifs play a significant role in limiting which features are significant at different phases of the stimulation. Moreover, estimating the information transferred by each signaling feature showed that in the majority of cases a single feature is insufficient to lead to a binary transcriptional decision (i.e. individual features carried less than one bit of information). In this light, decision tree analysis demonstrated that a combination of signaling features constitutes a feature based modulation code involving crosstalk in order to drive the cellular decision making. Given extensive single cell variability, our methodology is of practical utility to experimentalists who use single cell resolution data and are typically confronted with the variable aspects of the cellular activities [44, 45]. By focusing on dynamical features of the inputs to a signaling system, we have provided a novel framework which is broadly applicable to many signaling systems.

## Results

An integrated signaling dynamics and gene regulatory network model (Fig 1) was employed to bridge the variability between signaling dynamics and the transcriptional regulatory domain [40]. The regulatory network was modeled as ordinary differential equations using a combination of mass-action and Michaelis-Menten kinetics.

### Modeling dynamical features of transient signaling profiles

A phenomenological model for dynamics of a transient signaling kinase was developed to characterize independent time-invariant signaling features based on dynamical phases of delay, activation, peak retention and deactivation kinetics (Fig 2A; Methods section). An exponential ascent or decay model for temporal phases was derived to establish duration and time-constant features (see Table 1 for details). In order to capture observed variability in signaling dynamics, we introduced simultaneous variation in all of the identified upstream signaling features using a differential variation schema (Table 1 and S1B Fig). Our results revealed two distinct aspects of signaling variability. First, we observed that simultaneous variation in signaling features captured the variation of the entire signaling profile (shown as gray region in the Fig 2B, 2C and 2D). Second, these variations were found to be within experimental range measured from bulk tissue samples, shown as red error bars in the plots. Our results suggest that the variability in signaling features spans the combined effects of intrinsic and extrinsic fluctuations.

**Table 1:** List of independent signaling features characterized.

*Symbol*	*Feature Description*	*Characteristics*	*Value*	*Ref.*	*Variation*
**ERK**	**Extracellular-signal regulating kinase**			[93]	
*α*_1_E	Delay in activation	Duration	2		±2
*α*_2_E	Duration of activation	Duration	8		±4
*α*_3_E	Duration of peak	Duration	20		±4
*τ*_1_E	Time constant for activation	Time constant	1.5		× 2^±1^
*τ*_2_E	Time constant for peak retention	Time constant	17.5		× 2^±1^
*τ*_3_E	Time constant for deactivation	Time constant	5000		× 2^±1^
**FRK**	**Fos regulating kinase**			[94]	
*α*_1_F	Delay in activation	Duration	2		±2
*α*_2_F	Duration of activation	Duration	13		±4
*α*_3_F	Duration of peak	Duration	15		±4
*τ*_1_F	Time constant for activation	Time constant	3		× 2^±1^
*τ*_2_F	Time constant for peak retention	Time constant	130		× 2^±1^
*τ*_3_F	Time constant for deactivation	Time constant	20		× 2^±1^
**JNK**	**c-Jun n-terminal kinase**			[94]	
*α*_1_J	Delay in activation	Duration	5		±4
*α*_2_J	Duration of activation	Duration	25		±4
*α*_3_J	Duration of peak	Duration	15		±4
*τ*_1_J	Time constant for activation	Time constant	7		× 2^±1^
*τ*_2_J	Time constant for peak retention	Time constant	22		× 2^±1^
*τ*_3_J	Time constant for deactivation	Time constant	20		× 2^±1^

A combination of three distinct dynamical profiles for each signaling kinase ERK, FRK and JNK, respectively, was selected at random and simulations were performed for each of these combinations. A total of 100,000 simulations were conducted. The response of immediate early genes (IEGs) and transcription factors (TFs) resulting from these simulations revealed that variability in dynamical features propagates downstream as signal progresses in the network (gray regions in Fig 2E, 2F, 2G, 2H and 2I). Specifically, variability in downstream AP-1 TF, the aggregate of the two dimers, matched well with the observations from cell population measures [46], shown in red error bars, implying that variations in signaling features captures the biological variability at different levels in the network. Each dynamic signaling feature impacted the overall downstream response both positively and negatively, depending on the nature of the variations and dynamical interactions involved.

### Initial delay and activation kinetics of signaling dynamics control transcription factor dynamics during late phase

**Fig 3:**
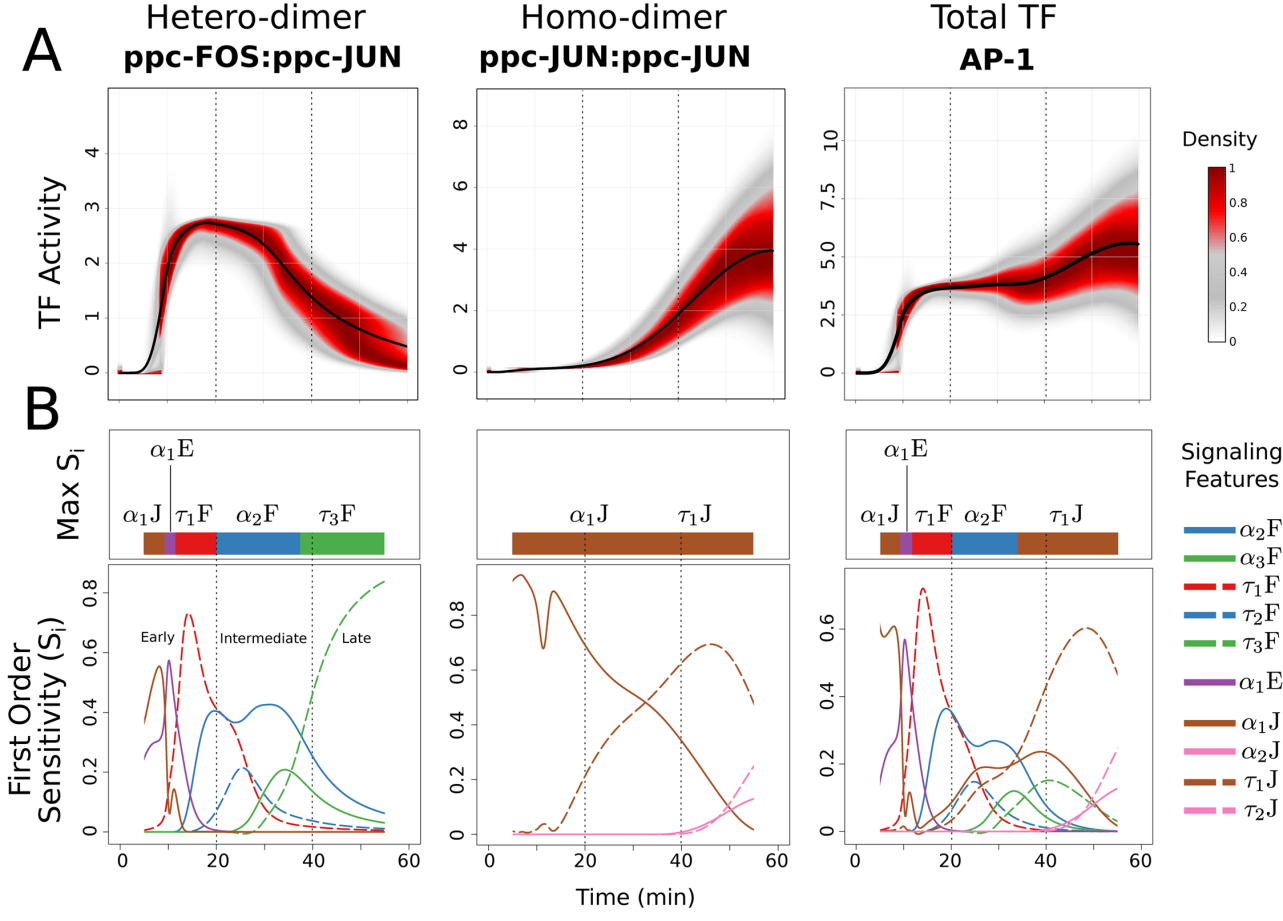
Transcriptional regulatory activity is controlled by initial delay and activation kinetics of the signaling dynamics. (A) Density plot for 100,000 simulation results at each time point from 0 to 60 minutes for transcription factors ppc-FOS:ppc-JUN, ppc-JUN:ppc-JUN and Total AP-1. (B) Variance based first order sensitivity coefficients (*S*_*i*_) of signaling features at each time point from 5 to 55 min as line plots to ppc-FOS:ppc-JUN, ppc-JUN:ppc-JUN and Total AP-1, respectively. The first and last five minutes of *S*_*i*_’s are not shown due to the expected instabilities in numerical estimation when dealing with near-zero values. Maximum *S*_*i*_ at each timepoint is shown as the color bar on top of each line plots. Refer to Table 1 for details of the signaling feature symbols.

Downstream response to receptor stimulation was divided into three phases: (1) Early phase where cells predominantly utilized the steady state TF proteins as well as triggered new IEG expression, (2) Intermediate phase where cells utilized new proteins transferred from IEG expression to modulate AP-1 TF composition and levels, and when the heterodimer ppc-FOS:ppc-JUN was the dominant contributor to AP-1 aggregate [47, 48], and (3) Late phase where ppc-JUN:ppc-JUN homodimer was the primary contributor to AP-1 TF [49]. It is important to note that transcription of c-Jun gene is slower in comparison to that of c-Fos gene [40], however, the translation process of c-Jun mRNA is much faster in comparison to that of c-Fos mRNA [41]. Variability in downstream TF dynamics (shown as density plots in Fig 3A) were decomposed to quantify the contribution of each signaling feature at different phases of the stimulation. Variance based global sensitivity analysis was performed to measure these contributions as first order and total sensitivities (see Methods). These evaluations revealed that AP-1 dynamics (both aggregate and individual dimers) were sensitive to only a select subset of signaling features at any given time (Fig 3B).

#### AP-1 response pattern was most sensitive to the initial delay in JNK activation, and to kinetics of FRK activation during early phase

Total AP-1, during early phase, was found to be sensitive to four signaling features, viz. delay in JNK activation (*α*_1_J), delay in ERK activation (*α*_1_E), duration and time-constant of FRK activation (*α*_2_F and *τ*_1_F, receptively) (Fig 3B). Heterodimer (ppc-FOS:ppc-JUN), during early phase, was sensitive to the same set of features as that of the Total AP-1, whereas homodimer (ppc-JUN:ppc-JUN) was sensitive to only delay in JNK activation (*α*_1_J). The only common feature that influenced Total AP-1 as well as the hetero/homodimers was *α*_1_J. This is consistent with the expectation based on network topology as c-JUN production is necessary to produce both the homodimer as well as the heterodimer. However, the remainder of the key features controlling Total AP-1 were also found to be key in controlling of the heterodimer response pattern. *α*_1_E showed a brief impact on heterodimer and AP-1 at around 10 minutes following the initiation of signaling dynamics (Fig 3B). However, the homodimer response showed a modest dip in first order sensitivity of the *α*_1_J feature due to higher order interaction between *α*_1_J and *τ*_1_J (revealed through higher order sensitivity indices in S3 and S4 Figs). *τ*_1_F was the most critical feature in shaping AP-1 activity dynamics at 20 minutes following the signaling stimulation, suggesting that the activation kinetics of FRK dominated the phosphorylation process of c-FOS proteins during the early phase.

#### Duration of FRK peak and kinetics of JNK activation were critical in shaping intermediate and late TF response dynamics

Total AP-1 was most sensitive to *α*_2_F during intermediate phase, but then became increasingly sensitive to *τ*_1_J during the late phase following the receptor stimulation (see the top color bar over Total AP-1 in Fig 3B). During the intermediate phase, the activity levels of heterodimer were dominant in terms of the proportion, which was also evident from the set of key features controlling Total AP-1 (*α*_2_F, *τ*_2_F, *α*_3_F and *τ*_3_F). JNK features (*α*_1_J and *τ*_1_J), which significantly influenced homodimer activity patterns, were also reflective in the critical features affecting the Total AP-1 response pattern during the intermediate phase. Importantly, the intermediate phase (20–40 minutes) revealed that the cellular information processing at the level of TFs involve signaling history, as indicated by the sensitivity of Total AP-1 as well as of the heterodimer to the dynamic signaling feature *α*_2_F. *α*_2_F represents activation phase of FRK from 0–18 minutes, which precedes intermediate response phase. Similar behavior was also observed in additional critical features to homodimer (*α*_1_J and *τ*_1_J during intermediate and late phases, respectively). In contrast, the most critical feature influencing heterodimer during the late phase showed that the kinetics of FRK deactivation dynamics (*τ*_3_F) were the key feature controlling the late heterodimer response pattern, thus revealing instantaneous information transfer between the signal and downstream response.

In the late response phase, Total AP-1 and the homodimer were sensitive to the same set of dynamic signaling features. This was expected, considering the dominance of homodimer in the composition of AP-1 during the late phase. However, the dynamical feature with the highest influence over the late phase of AP-1 dynamics was not instantaneously linked, but was a feature (*τ*_1_J) that preceded not just the previous phase, but two phases earlier to that of the late phase (i.e. early phase), revealing information transfer via history of signaling activity.

The dynamics of several critical features to Total AP-1, shown in Fig 3B closely resembled the predicted dynamics of activated transcription factor dimers. The aggregate TF showed a good resemblance to the predicted activation of hetrodimer by exhibiting similarity in critical features during the early phase, and then shifting to resemble the homodimer in the intermediate and late response phases. Functionally, delay in activation of the JNK (*α*_1_J) sensitivity pattern showed biphasic behavior, being critical in shaping the AP-1 during the very early phase, and then during the late phase (we explain this phenomena as the property of the regulatory network motifs in the next section). Interestingly, the first order sensitive pattern of *α*_1_J and *τ*_1_J that influenced homodimer activity pattern crossed each other during the intermediate phase, as homodimer took over the heterodimer as the major contributor to Total AP-1.

### Regulatory network motifs constrain information decoding

Transcription regulatory pathways contain a small set of recurring regulation patterns, called network motifs that are critical in processing of signaling information and in shaping of the response pattern. Our network contains two such recurring network motifs (S2 Fig): (1) FRK/ERK module showing a coherent feed forward interactions between receptor stimulated ERK pathway to activate ELK1 and subsequently transcribe IEG c-Fos, which is further activated (phosphorylated) by FRK to produce the heterodimer. (2) JNK module represents a positive feedback loop by expressing IEG c-Jun buffered via pp-ATF2:ppc-JUN dimer complex, and activation (phosphorylation) of the homodimer.

#### Feedforward interactions impose a bottleneck on information processing

**Fig 4:**
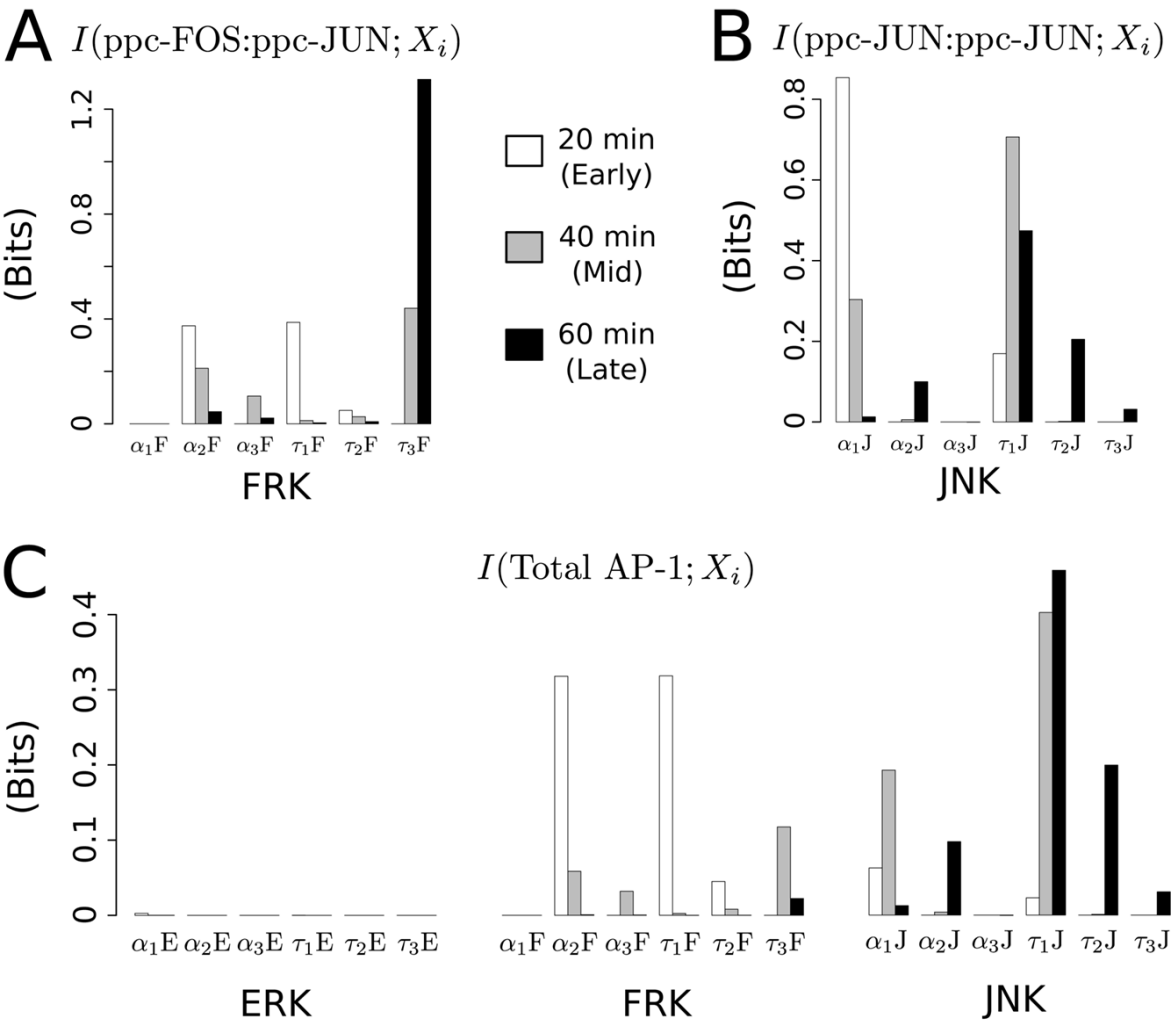
Information transduced by signaling features to downstream transcription factors. (A) Mutual information between each signaling feature at the snapshot measures of early (20 min as white), intermediate (40 min as gray) and late (58 min as black) phases to downstream transcription factor responses for ppc-FOS:ppc-JUN. (B) Mutual Information between signaling feature and ppc-JUN:ppc-JUN at different snapshot measures. (C) Mutual information between signaling features and Total AP-1. Only FRK and JNK signaling features transferred information to ppc-FOS:ppc-JUN and ppc-JUN:ppc-JUN, respectively, whereas features of both FRK and JNK signals transferred information to Total AP-1.

The ERK/FRK module was connected to activation of downstream TF ppc-FOS:ppc-JUN (heterodimer). The module contained a coherent feedforward loop [50] in which sufficient accumulation of c-FOS was necessary to instantiate the production of the heterodimer. The feedforward interactions required activation of both ERK and FRK in the production of the dimer, hence exhibiting ‘AND gating’ [28, 50]. ERK played an important role in the very early phase of expression of c-Fos by phosphorylating ELK1. But during the late phase, the rapid activation of heterodimer was constrained by the deactivation kinetics of the FRK. This is an integrative effect where the intermediate as well as the late phase of the dynamics were rate limited by the capacity of FRK to phosphorylate c-FOS (as revealed through the high sensitives to *α*_2_F, *τ*_2_F, *α*_3_F and *τ*_3_F in these phases). Since FRK was deactivating during the late phase, the kinetics of FRK deactivation (*τ*_3_F) was likely to be decoded at the level of the heterodimer. We test this possibility via estimation of mutual information as described below and illustrated in Fig 4.

#### Positive feedback loop introduces time lag in the information processing

In contrast to the feedforward module, the positive feedback module played a complementary role in maintaining the sustained dynamics of the Total AP-1. JNK dynamics activated as well as maintained phosphorylated c-JUN levels hence exhibiting a dual role in the regulation of the homodimer. As mentioned earlier, the slow rate of c-Jun expression resulted in the low rate of homodimer production during the early phase, but the dual effect of JNK activation generated a monotonic increase by localizing the dimer during the intermediate as well as the late phases, resulting in eventual dominance over the heterodimer activity levels after around 40 minutes. An ultrasensitive effect was observed where a very small amount of c-JUN protein in early phase resulted in a dramatic increase in the activity levels of the homodimer during the late phase. Therefore, the late phase dynamics of the homodimer downstream was determined by early phase of the JNK dynamics. The feature based sensitivity analysis revealed this non-intuitive result by quantitatively identifying kinetics of the JNK activation *τ*_1_J, as the most dominant feature during the late phase of the homodimer response, suggesting that history of the signaling dynamics was important in regulation of the transcriptional response. It further implicated that the downstream outcome was not only reflective of instantaneous observational change, but can be equally representative of the dynamical history of the upstream signaling activity.

### Individual signaling features carried insufficient information to lead to a downstream binary transcriptional regulatory decision

Estimating the lower bound of the Mutual Information (MI) between a signaling feature and downstream TF response revealed that majority of the information carried by individual features to TFs were less than one bit (See Methods and Fig 4). In this context, MI corresponds to the decoding of the TF level which can discriminate between the two levels of a dynamic signaling feature. Analysis of the information transfer at 20 minutes (early phase) revealed that *α*_2_F and *τ*_1_F transferred nearly equal amount of information (∼ 0.4 bits) to both heterodimer as well as to AP-1. In contrast, at 40 minutes (intermediate phase) and 60 minutes (late phase) the amount of information transferred by activation time-constant of JNK (*τ*_1_J) to AP-1 was the highest (∼ 0.4 and ∼ 0.45 bits, respectively). However, *τ*_1_J carried ∼ 0.7 and ∼ 0.5 bits of information to homodimer at 40 and 60 minutes, respectively, a complete reversal to the pattern observed in the Total AP-1. Importantly, the time-constant feature of FRK (*τ*_3_F) carried ∼ 0.4 bits at 40 minutes and ∼ 1.2 bits at 60 minutes, suggesting that the network has the capacity to decode this feature to make binary decisions at the later phase of the dynamic response. In contrast, the FRK time-constant *τ*_3_F carried less than 0.1 bit of information to Total AP-1. The signaling features of ERK had minimal information transfer to the TFs, in consistent with the network topology, as the effect of changes in ERK on the TFs is indirect and conditional on the dynamic features of FRK and JNK signaling.

### Combinations of activation and deactivation kinetics stratified transcription factor responses

**Fig 5:**
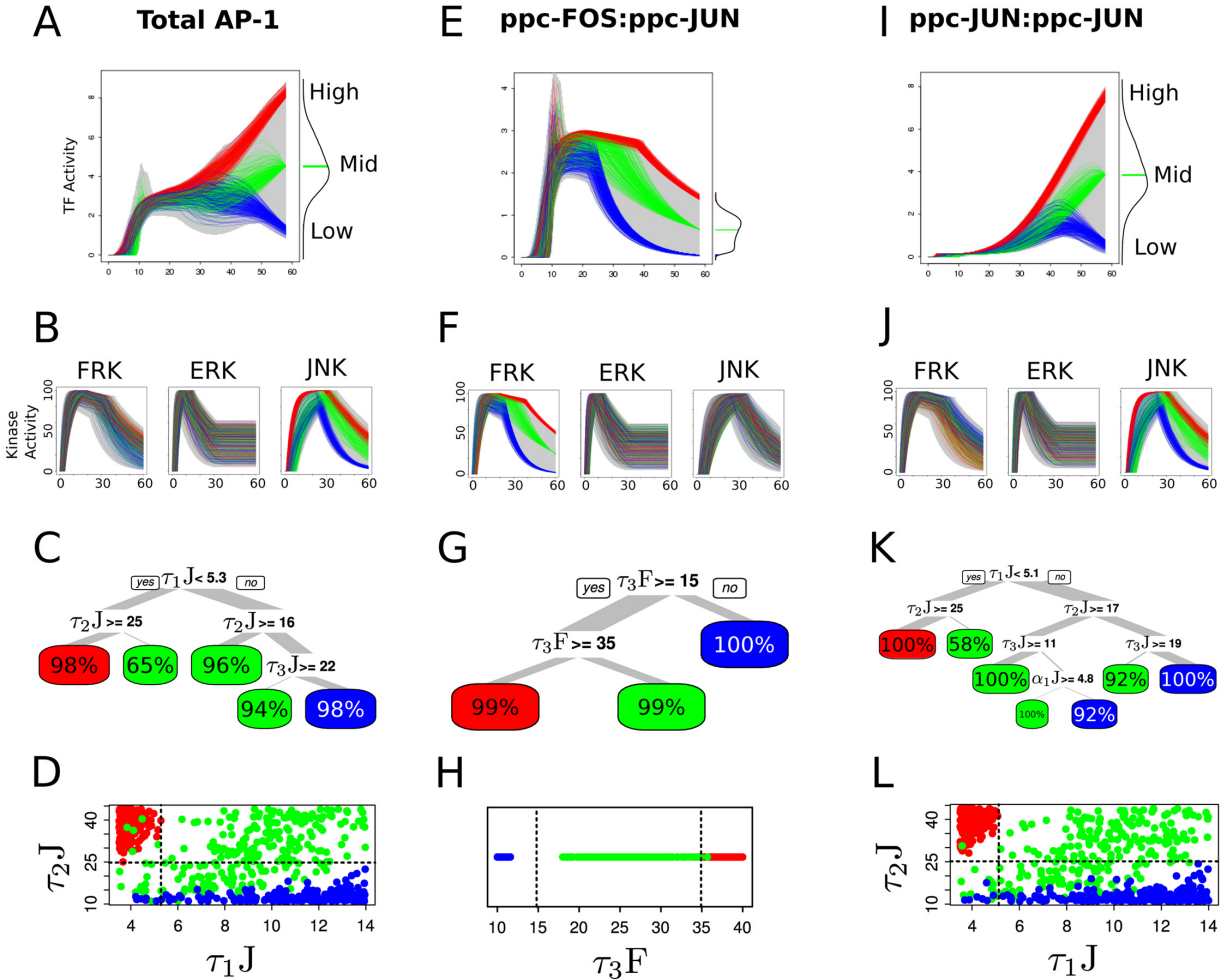
Combination of activation and deactivation kinetics of signaling dynamics stratify late response transcription factor phenotypes. (A) Late response (58 min) phenotypes for Total AP-1 transcription factor (TF) shown as High, Mid, and Low, represented by red, green, and blue colors, respectively. The red and blue phenotypes were selected from upper and lower margins of the distribution at the late time point, respectively (distribution is illustrated on the right side of the figure), and mid phenotype profiles where selected from the median region of the distribution. The first 250 profiles are shown for each response TF phenotype, selected from their representative statistical regions. The gray background corresponds to spanning of the 100,000 simulations resulting from a dense simultaneous randomization of signaling features (see Methods section for details). (B) The corresponding upstream signaling kinase profiles for FRK, ERK and JNK responsible for shaping the Total AP-1 response TF phenotypes displayed in A. (C) Decision tree displaying the combination rules of signaling features essential to stratify late Total AP-1 downstream response TF phenotypes (red, green and blue). The tree root node represents the most dominant feature, the next branch nodes represent relatively less dominant features, and so on. The width of the tree branch represents the number of cells, and the percentage in each leaf constitutes the proportion of those cells belonging to the respective phenotype, represented via red (High), green (Mid) or blue (Low) color. (D) Scatter plot of the first two dominant signaling features distinctively separating the three Total AP-1 phenotypes in feature-space, shown through dotted lines. These dotted lines are the classifiers estimated by the root, and the first significant branch of the decision tree in C. *τ*_2_J and *τ*_1_J splices out Total AP-1 late response phenotypes for Total AP-1. (E) Late response phenotypes for ppc-FOS:ppc-JUN TF, shown as red, green and blue. See A for details. (F) The corresponding 250 signaling kinase profiles which shaped ppc-FOS:ppc-JUN TF phenotypes. (G) Decision tree displaying the combination rules of signaling features necessary to stratify the late ppc-FOS:ppc-JUN downstream responses. See C for figure details. (H) Plot showing dominant signaling feature, *τ*_3_F, distinctively separating three ppc-FOS:ppc-JUN phenotypes. (I) Late response phenotypes for late ppc-JUN:ppc-JUN response, shown as red, green and blue, respectively. See A for figure details. (J) The corresponding signaling profiles that shaped ppc-JUN:ppc-JUN TF phenotypes. (K) Decision tree displaying the combination rules of signaling features necessary to splice late ppc-JUN:ppc-JUN downstream phenotypes. See C for details. (L) Scatter plot of the first two dominant signaling features, *τ*_1_J and *τ*_2_J distinctively separating three ppc-JUN:ppc-JUN phenotypes in their feature-space, shown via dotted lines.

We extended our results on global sensitivity analysis and information transfer to study whether the variable responses at the single cell level could be analyzed for inferring the dynamic features of upstream signaling. We considered a typical case involving snapshot measures from single cells, e.g., gene expression levels, or immunocytochemistry data on activated TF protein levels [51]. Decision tree analysis was performed (see Methods) on downstream TF activity phenotypes at three different timepoints: 20 minutes (S5 Fig), 40 minutes (S6 Fig) and 60 minutes (Fig 5). We observed that the TF activity response at any given time point was a distribution, and a function of varying upstream signaling features. Three distinct phenotypes at each time point were defined as High, Mid, and Low TF levels (Fig 5). The High and Low phenotypes represent the upper and lower margins of the distribution, respectively, and the Mid phenotype corresponds to the median of the distribution. We considered the TF phenotypes of Total AP-1 (Fig 5A), heterodimer ppc-FOS:ppc-JUN (Fig 5E), and homodimer ppc-JUN:ppc-JUN (Fig 5I). Mapping these TF phenotypes to corresponding kinase profiles resulted in distinguishable signaling features that would yield distinct TF response dynamics (Fig 5B, 5F and 5J). The combination of key features varied depending on the specific TF phenotype analyzed: JNK features appear to be distinct for the Total AP-1 and homodimer phenotypes (Fig 5B and 5J), whereas FRK features were specific to the stratification of the heterodimer phenotypes (Fig 5F). We developed decision trees to organize these signaling features into a hierarchy of rules defining the ranges of variation that stratify activation profiles of downstream TF phenotypes.

For the Total AP-1 TF snapshot response measure at late phase, the decision tree analysis revealed a hierarchy of rules based on three signaling features of JNK (*τ*_1_J, *τ*_2_J and *τ*_3_J) as primarily yielding downstream phenotypes (Fig 5C). Our results revealed that fast activation of JNK (*τ*_1_J < 5.3) and very slow peak deactivation of JNK (*τ*_2_J > = 25) were highly likely to drive cells to a High AP-1 phenotype, even in presence of variations in other kinase signaling features. In contrast, slow activation of JNK (*τ*_1_J >= 5.3), fast peak-deactivation of JNK (*τ*_2_J < 16), and fast deactivation of JNK (*τ*_3_J < 22) were very likely to drive cells to Low AP-1 phenotype. We assessed the decision tree analysis results in an alternative visualization layout considering the top two dominant features from the decision trees in a scatter plot, and mapped the rules as well as the phenotype categories to the plot to observe how these features separated cells with a specific phenotype (Fig 5D). The extremes, High and Low AP-1, were well separated by specific intervals of *τ*_1_J and *τ*_2_J, with faster activation and slower inactivation leading to a High AP-1 phenotype. In the case of heterodimer phenotypes, *τ*_3_F was found to be the only dominant feature separating all of the three phenotypes (Fig 5G and 5H). On the contrary, the homodimer phenotypes were segregated by a combination of *τ*_1_J, *τ*_2_J, *τ*_3_J and *α*_1_J (Fig 5K and 5L).

The results from decision trees were in agreement with those from analysis of MI. Features which carried one or more than one bit of information to downstream TF responses were sufficient to individually distinguish High, Mid, and Low phenotypes. For example, *α*_1_J separated homodimer phenotypes at 20 minutes (S5K and S5L Fig), and *τ*_3_F separated heterodimer phenotypes at 60 minutes (Fig 5G and 5H). *α*_1_J carried ∼ 0.85 bits of information to homodimer at 20 minutes, and *τ*_3_F carried ∼ 1.2 bits of information to heterodimer at 60 minutes (Fig 4). In summary, decision tree analysis revealed that an individual feature which transferred ≥ 1 bit of information corresponds to the distinct downstream TF phenotypes, with potential consequences for fine tuning cellular decision making in response to external stimuli.

#### Immediate early gene expression profiles were controlled by the kinetics of peak retention

**Fig 6:**
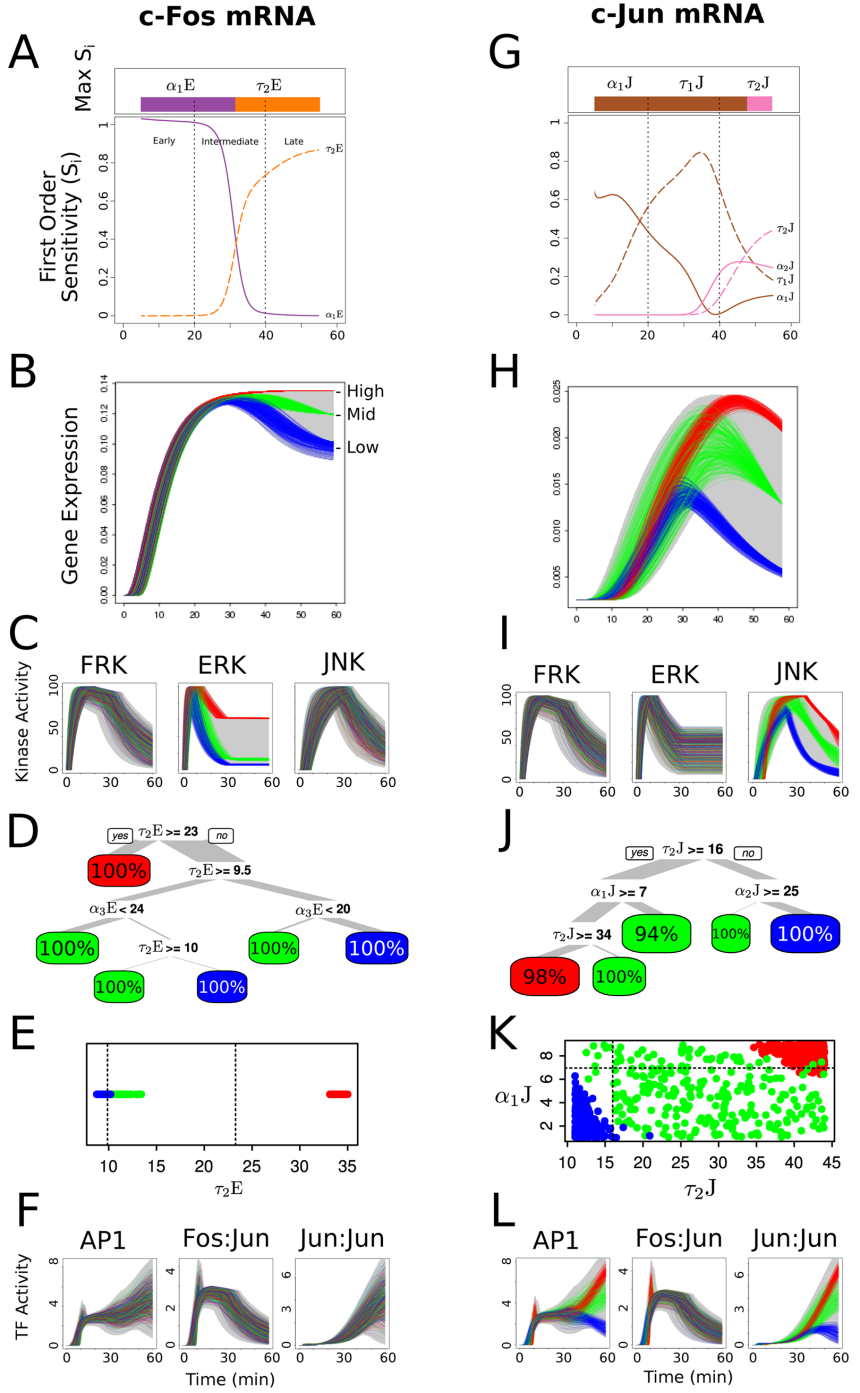
Activation kinetics of peak retention controls the late response of the immediate early gene expression dynamics. (A) First order sensitivity indices at each timepoint from 5 to 55 min for c-Fos mRNA. See Fig 3B for details. (B) High, Mid and Low c-Fos immediate gene expression (IEG) phenotypes at late phase of the dynamics as shown by red, green and blue colors, respectively. See Fig 5A for details. (C) The corresponding upstream signaling kinase profiles for FRK, ERK and JNK responsible for shaping the c-Fos IEG phenotypes displayed in B. (D) Decision tree displaying the combination rules of signaling features necessary to stratify the late c-Fos IEG downstream responses. See Fig 5C for details. (E) Plot of the only dominant signaling feature, *τ*_2_E, distinctively separating three c-Fos IEG phenotypes. (F) The corresponding downstream transcription factor profiles for Total AP-1, ppc-FOS:ppc-JUN and ppc-JUN:ppc-JUN shaped by c-Fos IEG phenotypes displayed in B. (G) First order sensitivity indices to c-Jun mRNA. (H) High, Mid and Low c-Jun IEG phenotypes at late phase of the dynamics (I) The corresponding upstream signaling kinase profiles for FRK, ERK and JNK responsible for shaping the c-Jun IEG phenotypes, displayed in H. (J) Decision tree displaying the combination rules of signaling features necessary to stratify the late c-Jun IEG. (K) Scatter plot of the first two dominant signaling features, *τ*_2_J and *α*_1_J distinctively separating three c-Jun IEG phenotypes, shown through dotted lines. (L) The corresponding downstream transcription factor profiles shaped by c-Jun IEG phenotypes, displayed in H.

Recent technologies have enabled gene expression assays at single cell scale with unprecedented resolution, and at a very high throughput with hundreds of samples [20, 22, 52]. We have recently demonstrated that the variations and patterns in the gene regulation in single cells correspond to their cellular inputs [20]. A limitation of these single cell assays is that they provide a static snapshot of the gene expression levels at one timepoint. We examined whether these snapshot measure can be used to infer the dynamic features of the upstream signaling. Through global sensitivity analysis, we evaluated the key features that controlled the gene expression levels of c-Fos and c-Jun in the network. Delay in activation of ERK (*α*_1_E) during the early phase, and time-constant of peak retention of ERK (*τ*_2_E) in later phases were the dominant signaling features that shaped c-Fos expression (Fig 6A and 6B). In contrast, delay in activation of JNK (*α*_1_J) during the early phase, and time-constant of JNK activation (*τ*_1_J) and JNK peak retention (*τ*_2_J) during the intermediate and late phases, respectively, were dominant features in defining the c-Jun expression pattern (Fig 6G and 6H). Considering the slow rate of transcription in comparison to that of the translation rate of c-Jun, the mRNA levels attain their peak during intermediate phase, but start to decrease during the late phase. The stability of c-Fos mRNA ensures a sustained peak level during the late response phase in addition to the slow rate of ERK deactivation. Interestingly, none of the deactivation features influenced gene expression. The delay in activation (*α*_1_) of signals appeared to have significant effect during early phases while the time constant of peak retention (*τ*_2_) controlled gene expression during the late phase.

We considered which signaling features shape High, Mid and Low expression levels, and how do these levels corresponds to the downstream TF response patterns? The decision tree analysis revealed that the time constant of ERK peak retention (*τ*_2_E) was the key distinguishing feature for the c-Fos gene expression phenotypes (Fig 6C, 6D and 6E), whereas a combination of the time constant of JNK peak retention (*τ*_2_J) and duration of JNK activation (*α*_2_J) were the key features in distinguishing the c-Jun gene expression phenotypes (Fig 6I, 6J and 6K). We further analyzed how individual IEG levels distinguish downstream TFs (Fig 6F and 6L). Our results show that at late phase, c-Fos expression levels have no significant impact on TF levels, while c-Jun levels were likely to stratify homodimer and Total AP-1 response patterns.

### Variability in the kinetics of peak retention drives variability in immediate early gene expression pattern in single cells

**Fig 7:**
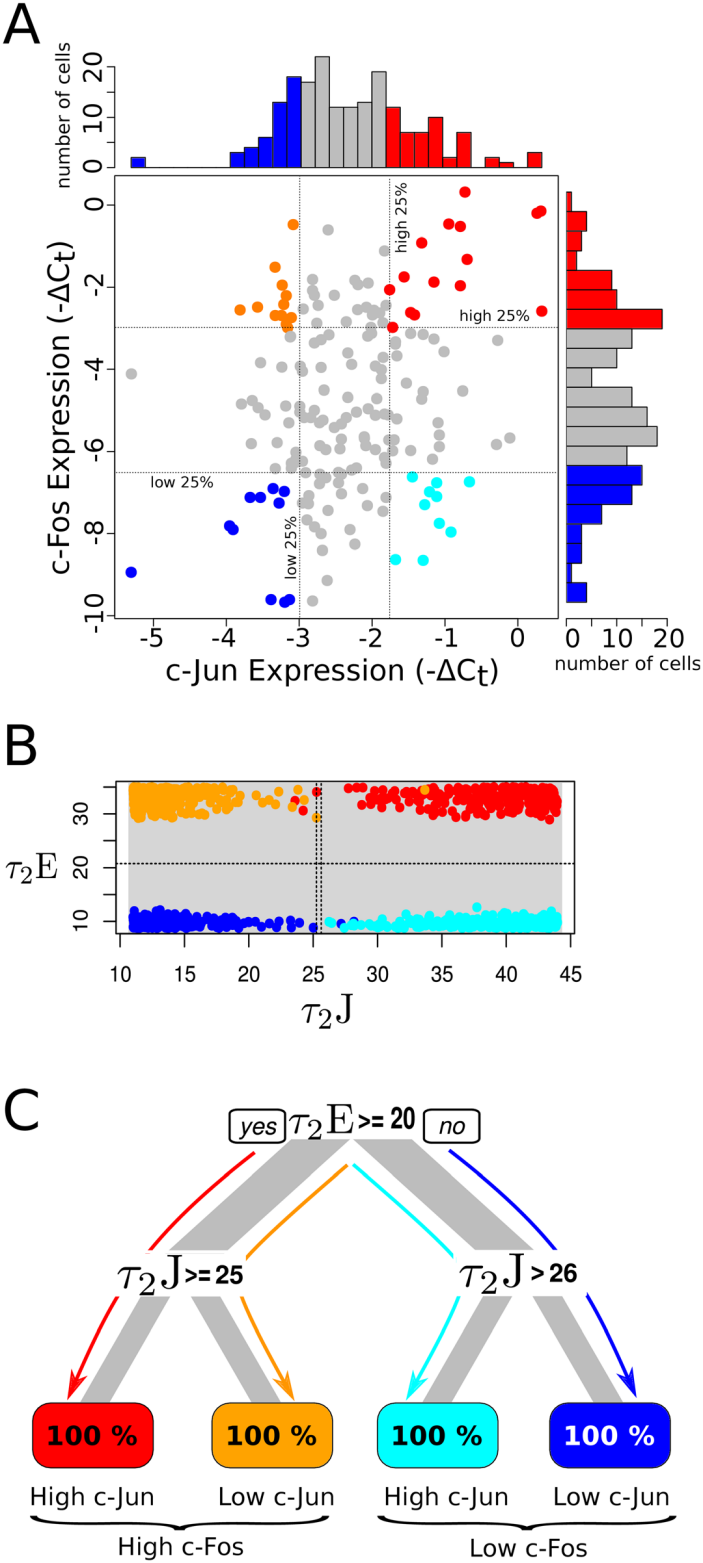
Kinetics of the peak retention of upstream signaling stratify late response of immediate early gene expression patterns in single cells. (A) Scatter plot showing bivariate immediate early genes c-Fos and c-Jun expression levels in individual cells picked from same cell types (data from [20]). Expression levels (−ΔC_*t*_ measured by realtime PCR) of 151 single cells are shown at 60 min after an induced hypertension stimulus (refer [20] for details of the experiment). Marginal histograms for 151 cells are shown at the top and right for c-Jun and c-Fos IEG, respectively. Four phenotypes were statistically determined from top 25 percentile and bottom 25 percentile of c-Fos and c-Jun expression distributions, respectively. Red corresponds to ‘high c-Fos and high c-Jun’ phenotype, cyan corresponds to ‘low c-Fos and high c-Jun’ phenotype, blue corresponds to ‘low c-Fos and low c-Jun’ phenotype, and orange ‘high c-Fos and low c-Jun’ phenotype. (B) Scatter plot of the feature-space for the first two dominant signaling features, *τ*_2_J and *τ*_2_E, distinctively separating four gene expression phenotypes, determined through similar statistics from model simulations. Note that the colors are same as that of the respective phenotypes defined in A. The first 250 cells in each phenotypic region were used in the analysis. The dotted lines in the plots are the classifiers estimated by the root, and the next significant branches of the decision tree in C. The gray region in the background corresponds to the variability in 100,000 cells, spanning the entire functional space of the signaling features. (C) Decision tree analysis to identify the key features and their conditionalities driving the downstream immediate early genes expression phenotypes as the categorizers of upstream features, for the cells in B. See Fig 5C for details.

We further analyzed bivariate gene expression patterns of immediate early genes (c-Fos and c-Jun) as observed in single cell experiments (Fig 7A, data is from [20]). Despite the cells being picked from a pool of neurons of same type under homogeneous conditions, experiments show that the variability in the gene expression levels is extensive [53] (almost 4-8 fold variation in the abundance). In order to identify the key upstream signaling features that lead to distinct phenotypes of the observed response distributions (Fig 7A), we employed a model based approach to analyze statistically similar distribution of the single cells from our simulation results. Four groups were identified based on the high and low 25^th^ percentile of the marginal distributions: (1) high c-Fos, high c-Jun, (2) high c-Fos, low c-Jun, (3) low c-Fos, high c-Jun, and (4) low c-Fos, low c-Jun. We found that the key controlling features established from our earlier analysis (Fig 6), separated the four IEG groups distinctively in the signaling feature space (Fig 7B). Decision tree analysis (Fig 7C) revealed that: (1) slow rate of ERK peak deactivation (high *τ*_2_E) and slow rate of JNK peak deactivation (high *τ*_2_J) produced high c-Fos and high c-Jun gene expression pattern, (2) slow rate of ERK peak deactivation (high *τ*_2_E) but fast rate of JNK peak deactivation (low *τ*_2_J) produced high c-Fos and low c-Jun gene expression pattern, (3) fast rate of ERK peak deactivation (low *τ*_2_E) but slow rate of JNK peak deactivation (high *τ*_2_J) produced low c-Fos and high c-Jun gene expression pattern, and (4) fast rate of ERK peak deactivation (low *τ*_2_E) and fast rate of JNK peak deactivation (low *τ*_2_J) produced low c-Fos and low c-Jun gene expression pattern. Our results indicate that the variability is the gene expression patterns in single cells is likely to contain information on features of the upstream signaling dynamics.

### Patterns of sensitivity and information transfer map correlates with topological organization of the network modules

**Fig 8:**
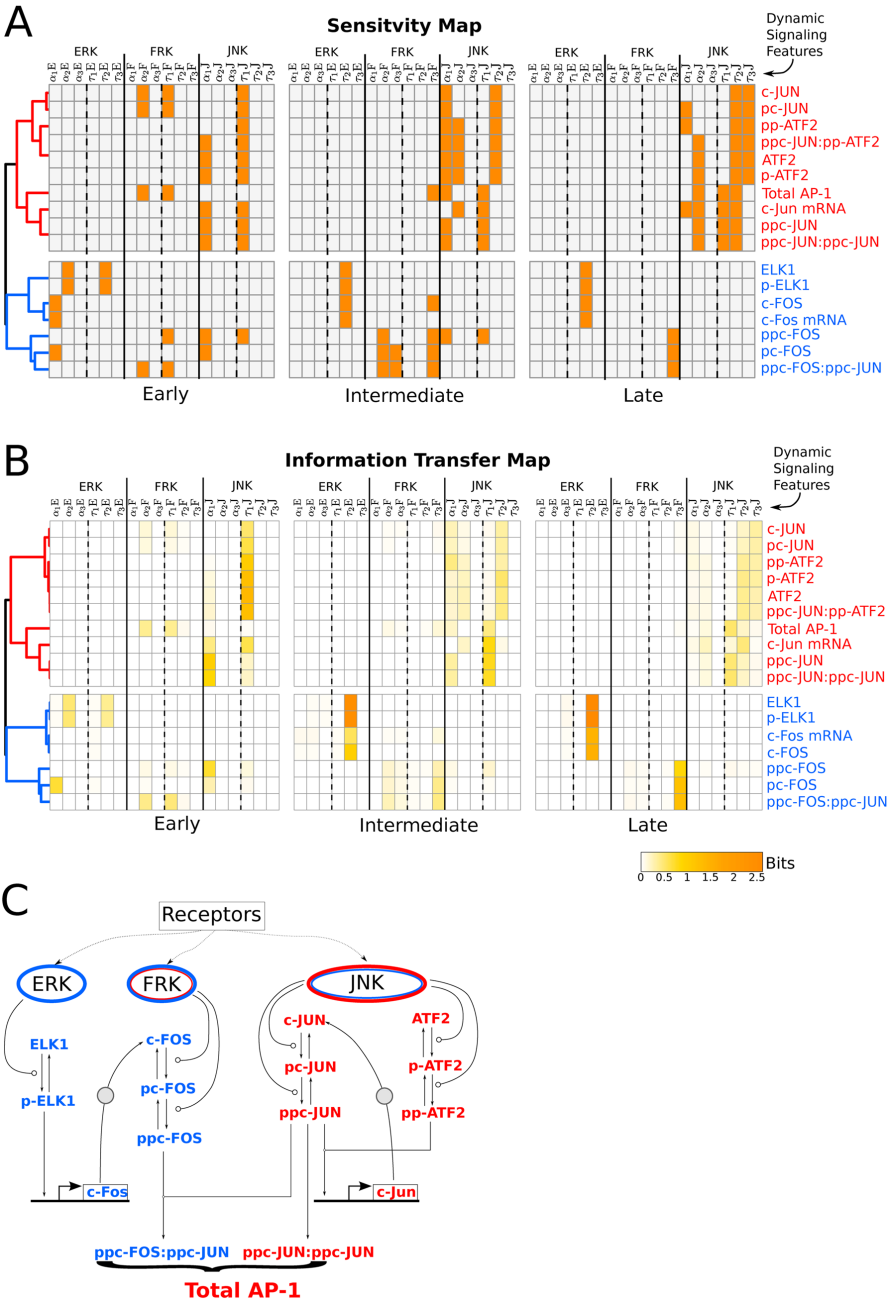
Patterns in sensitivity and information-transfer maps correlate with topological organization of the network modules. (A) Heatmap of sensitive signaling features to each intermediary response species at snapshot measures of early (20 min), intermediate (40 min), and late (58 min) phases of the stimulation. Orange colored boxes in the map represents sensitive features (*S*_*i*_ > 0.1), and gray color represents non-sensitive features(*S*_*i*_ ≤ 0.1). Unsupervised hierarchical clustering (of Pearson correlation distance matrix) was performed on the heatmap, shown as dendrograms on the left. Clustering unwinds coherent feedforward and positive feedback network motifs shown as blue and red colored species, respectively. (B) Heatmap of mutual information between each feature and intermediary response species for snapshot measures at early (20 min), intermediate (40 min) and late (58 min) phases of the stimulation. The scale bar reports the estimated mutual information in bits. Unsupervised hierarchical clustering (Pearson correlation distance matrix) was performed on the heatmap shown by dendrograms on the left. (C) Gene regulatory network showing network motifs of feedforward interactions (ERK/FRK module) and positive feedback loop (JNK module) as blue and red, respectively.

Our analysis thus far revealed that specific features of signaling dynamics correspond to the patterns of downstream gene regulation and TF activity. The results also indicated that distinct signaling features can be decoded depending on the downstream location in the regulatory network. This motivated us to consider whether there was a higher order organization of which features being decoded and where in the network. We performed exhaustive global sensitivity analysis and information transfer assessment to consider each node in the network as the output of interest. For each node, we computed the sensitivity indices corresponding to the dynamic signaling features as well as the information transfer between the signaling feature at the corresponding node levels. We represented the results as a Sensitivity Map and Information Transfer Map. The Sensitivity Map lays out all of the controlling features (*S*_*i*_ > 0.1) for different nodes in the regulatory network and at different phases of the stimulation (early, intermediate and late) (Fig 8A). Similarly, the Information Transfer Map lays out the extent of information transferred by each feature to different network decoders (mutual information between signaling features and the downstream responses) and at different phases of the stimulation (Fig 8B). Unsupervised clustering of these maps yielded two major groups corresponding to the two regulatory motifs that drove distinct downstream activity patterns: transcriptional feedforward and transcriptional positive feedback interactions (Fig 8C). Both of these maps independently separated regulatory motifs along the network decoders thereby revealing the interdependence of information processing by signaling features and by network topology.

## Discussion

We present a new paradigm in which dynamic signaling features and not molecular abundances are introduced as the carriers of functional information. We developed a novel empirical model for signaling profiles based on transient phases of activation, peak retention and deactivation kinetics to characterize the dynamical aspects of the signal. We then performed dense Sobol sampling over an uniform high-dimensional space of signaling features to map the functional space of signaling dynamics which spans all possible essential dynamical profiles for three upstream signaling kinases, namely ERK, FRK and JNK respectively. We employed variance based global sensitivity analysis, estimated mutual information, and developed decision trees to identify the key controlling features. The amount of the information carried to various network components were also analyzed, specifically to the IEG and TF responses. Our results showed that the key controlling feature must carry at least one bit of information in order to distinctively separate downstream IEG and TF dynamical response patterns. Our analysis revealed that in the majority of cases an individual signaling feature carried insufficient amount of information (less than one bit) to directly lead a binary transcriptional regulation, and therefore a combination of features influenced downstream regulatory process. Cellular decision, hence, is a consequence of decoding of multiple dynamic signaling features, rather than a direct function of the abundances of functionally active signaling effectors.

With recent advancements in single cell technologies such as fluorescent labeling techniques, optogenetics, gene-expression profiling, and time-lapse microscopy, it is evident that cells transduce functional information through time-dependent activity changes in one or more signaling molecules [9–11, 54–56]. For example in a recent single cell quantitative live-cell imaging study, it was found that individual macrophages exhibited analog responses to LPS-induced NF-*κ*B dynamics [57], in contrast to the digital responses previously reported in cytokine treated non-immune cells [58, 59]. Cells, therefore, are capable of interpreting a stimulus in a continuous manner for which the time-dependent aspect of the signal represents an embedded functionality. We explored a scheme where the cell’s signaling code involves dynamic signaling features. These features represent different kinetic modulation of the signal as the functional aspect of a molecular activity change. Simultaneous variation in these features yielded signaling dynamics as variable as observed experimentally, and also bounded within the limits of the population measure. As the signal progressed in the network, the variability through dynamical features also progressed resulting in a non-uniform response distribution downstream, in consistent with other studies [24, 26, 60, 61]. Our results showed that this downstream response distribution were also within the biological variability reported in the population response.

Using global sensitivity analysis, decomposition of the variance of the downstream IEG and TF responses revealed that the response distributions were essentially a result of multiplexing of various signaling features. Our results implied that pleiotropic regulators such as signaling pathways are likely to employ a feature-based modulation code to transduce intracellular information and elicit context-specific responses. This code includes combinations of time-invariant signaling features including duration, time constant, amplitude and/or frequency of dynamics to ensure specificity in the downstream responses. Further, our approach employs decision tree analysis to identify the rules involving upstream signaling features to understand the nature of decision making, enabling us to unravel the complexity of cellular information processing. Our approach points to a novel modulation code involving dynamic features of signaling, through which the gene regulatory phenotypes may be shaped. In such a formulation, the manipulation of signaling towards desired gene regulatory responses involve alterations in the signaling onset, duration, etc [62]. This is in stark contrast to an abundance based code that would use over/under-expression or knock in/out based interventions. Analyzing the implications of transcriptional variability at the single cell level must include the interplay between the properties or features of the upstream signaling dynamics [41, 60]. Decision tree analysis allow us to establish the functional implications of variability in signaling dynamics in cellular decision and elucidate how this variability may result in cellular (or phenotypic) heterogeneity.

Our results revealed that correlations between activity levels of signaling molecules and downstream responses are insufficient to establish a physiologically relevant code. For instance AP-1 activity levels were found to be sensitive to a different subsets of signaling features at different phases of the stimulation. Our analysis found that the regulatory network is as essential an element in cellular decision making. Intracellular information decoding is equally as critical as information encoding. Similar observations have been reported in a single cell study where dual reporters were used to characterize live-cell response through LPS induced NF-*κ*B dynamics, and to learn how they correlated with inflammatory cytokine gene-expression output [57]. When the instantaneous ratio of abundance levels was calculated, the transcriptional regulator (NF-*κ*B) and downstream transcription (TNF-*α* gene reporter) were poorly correlated. An unbiased regression analysis of NF-*κ*B dynamics over entire stimulation time course was found to be the key determinant of the transcriptional output, suggesting the limitation of comparing molecular abundances. In the same study, it was observed that the macrophages showed a robust discrimination to different LPS doses. This was because the information decoding involved a feedback dominance switching which stratified the immune response despite the limited information encoding capacity of the signaling network [17]. Hence, our dynamic feature based information processing framework can be readily employed to study cellular decision making in a broad range of biological contexts.

Variability in signaling dynamics [63] and in transcriptional regulation [25, 41] have been typically studied in isolation of each other. For instance, studies on the dynamics of ERK2 translocation in single human cells reveal a large variation in basal ERK2 nuclear levels [63, 64]. Besides peak fold change, other features of the dynamic response pattern such as final fold change, the peak delay and timing of activation also exhibit high variability [65]. Similar variability is also seen among the single cell time courses of NF-*κ*B dynamics treated across the range of LPS concentrations [57]. On the other hand, the seminal studies of transcriptional variability in bacteria and yeast have demonstrated that inherent stochastic noise in gene expression (intrinsic variability), as well as variability in cellular transcription factor activity levels (extrinsic variability), can result in cellular heterogeneity [26]. We have also reported similar observations in mammalian cells where cell-to-cell variability of transcriptional change was shaped by the inputs to the cells, suggesting the patterns of response distribution are based on a systematic processing of inputs instead of being a noise around the mean [20]. Our framework bridges the variability between the signaling dynamics and transcriptional regulatory responses through a model based approach. Most interestingly, our results demonstrate that even a single snapshot response measure of single cell transcriptional activity can be informative of upstream signaling dynamics. Further analysis revealed that different network components (IEG vs TF) decode different aspects of the signaling dynamics, indicating that a network component can only decode a portion of the signaling history. For instance, AP-1 response dynamics were informative of a combination of activation and peak retention kinetics of the JNK signal, whereas c-Fos and c-Jun IEGs were informative of peak deactivation kinetics of ERK and JNK signals, respectively. This distinction in dynamic decoders is a result of multiplexing of signaling features via regulatory network motifs. Feedforward interactions imposed a bottleneck to information processing, where early phase features such as delay and activation kinetics of FRK did not transmit information to downstream TFs. In contrast, positive feedback loop introduced a time lag in information processing where the late phase of the downstream AP-1 TF was informative of activation kinetics (early phase) of the JNK signal. Other studies on signaling dynamics, particularly those considering networks with feedback and feedforward loops, use much longer term analysis than just one hour [66, 67]. Opportunities exist for longer-term dynamics to unravel additional complexities of these regulatory network motifs which may not be captured by shorter-term dynamics.

Studies to date have analyzed the mechanism of cellular decision making as related to dynamics of signaling activities by evaluating the correlation between the upstream and downstream molecular abundances. For instance, high correlation between EGF binding to EGFR and subsequent cell proliferation [64], or high correlation between increase in IL2 and IL4 receptor trafficking and consequent T cell proliferation [68, 69] have been interpreted as information transfer from the activated receptor levels to the downstream effector activities. However, inferences based on instantaneous correlations do not consider how cells process the time-dependent activity changes. The implicit assumption to these studies is that instantaneous change in molecular abundances between the upstream and downstream events is the natural mechanism of cellular information encoding [31, 70]. Such an approach may establish a causality between the two domains, but limits our understanding of any functional capabilities that could be gained by processing dynamical pattern. Our approach of reformulating the problem overcomes these limitations for a novel analysis of cellular information processing that takes dynamics into account.

Emerging single cell studies are illustrating the use of high temporal resolution data sets to unravel the mechanism underlying cellular decision making process. For instance in a recent study, a microfluidic setup was used to dynamically administer inhibitor treatment to alter the amplitude, frequency, and duration of nuclear localization of transcription factor Msn2, while a fluorescent reporter of Msn2 transcriptional activity was used to detect the downstream response [7, 33]. The study revealed that different expression patterns correlated with distinct dynamical features of administrators. As another example, a short duration of LPS stimulus does not elicit an immune response, whereas persistent LPS stimulus yields an innate immune response [71]. Such results are consistent with our findings that the information transfer underlying cellular decision making involves dynamic aspects of signaling. We found that the dynamic decoding is in close alignment with the network topology, as revealed by unsupervised clustering of the newly developed Sensitivity and Information Transfer Maps. Our findings suggest a novel dynamic modulation code, where signaling features in coordination with the network motifs shape the downstream regulatory patterns. Our approach provides a novel framework to study the structural features, i.e., static aspects of a network, in integration with signaling and transcriptional regulatory kinetics, i.e., dynamic aspects of the function, to understand information transfer underlying cellular decision making process. Understanding these dynamic encoding-decoding principles would enable sophisticated strategies to manipulate signaling dynamics to better control cellular responses in a broad range of biological contexts.

## Methods

### Integrated model of signaling dynamics and gene regulatory networks

We defined gene regulatory model as a combination of regulatory network ℱ and external input signal *f*_*s*_ as

$$\begin{array}{c} \dot{y}=F(t,y_{0},k,f_{s}\left(X,t\right)) \end{array}$$ (1)

where species $\text{y}\in {\mathbb{R}}^{s}$, parameters $\text{k}\in {\mathbb{R}}^{m}$ and signaling features **X**.

### Gene regulatory network

Activation of receptor due to ligand binding on the cell surface elicits and localizes multiple signaling kinases [72]. In our model, extracellular-signal-regulated kinase (ERK), c-FOS regulating kinase (FRK) and c-Jun N-terminal kinase (JNK) were activated in cells through receptor stimulation, to transcriptionally and post-translationally regulate the activator protein-1 (AP-1) family of TFs (See [40, 73] for details on model development). Family of AP-1 TFs considered here are: the heterodimer ppc-FOS:ppc-JUN and the homodimer ppc-JUN:ppc-JUN. These activated AP-1 TFs subsequently bind to promoters of key target genes resulting in gene expression changes [20, 74]. Activation of target genes is involved in regulation of blood pressure and the development of hypertension in brainstem [74–76]. Hence, AT1R stimulated multiple signaling kinases in brainstem neurons are tightly coupled with the specific physiological function of controlling blood pressure. This mechanistic model was implemented as a set of ordinary differential equations that integrated three signaling kinases viz. FRK, ERK and JNK with activation of AP-1 TFs (Fig 1). Experimentally measured time course activity change in three kinases were interpreted as input signals [40, 46], and subsequent changes in the activity of IEGs and TFs as the downstream responses [40, 77]. Details of the model equations are reported in S1 and S2 Tables. The model code in MATLAB format is available through accession No. 185122 on the ModelDB resource [78] at https://senselab.med.yale.edu/ModelDB/showModel.cshtml?model=185122.

### Characterizing dynamical signaling features

In our model, signaling kinase profiles were assumed to be an outcome of enacting activation, peak retention, and deactivation processes, initiated in parallel by receptor stimulation through ligand binding (input stimulus) [79, 80]. A simplified phenomenological interpretation of the phosphorylated kinase signaling profile can then be developed as the difference between first order activation and peak processes, and both of them opposed by a first order deactivation processes, represented as:

$$\begin{array}{c} f_{s}\left(t\right)=(z_{1}\left(t\right)-z_{2}\left(t\right))-z_{3}\left(t\right) \end{array}$$ (2)

where *f*_*s*_ is the dynamics of the signaling kinase, *z*_1_ are the activation processes, *z*_2_ are the peak processes, and *z*_3_ are the deactivation processes initiated by a receptor stimulus as shown in control diagram (S1A Fig).

$$\begin{array}{ccc} \dot{z_{1}}\left(t\right) & = & k_{a}^{z_{1}}u\left(t\right)-k_{d}^{z_{1}}z_{1}\left(t\right) \end{array}$$ (3)

$$\begin{array}{ccc} \dot{z_{2}}\left(t\right) & = & k_{a}^{z_{2}}u\left(t\right)-k_{d}^{z_{2}}z_{2}\left(t\right) \end{array}$$ (4)

$$\begin{array}{ccc} \dot{z_{3}}\left(t\right) & = & k_{a}^{z_{3}}u\left(t\right)-k_{d}^{z_{3}}z_{3}\left(t\right) \end{array}$$ (5)

where *u*(*t*) is a function representing the upstream stimulus, $k_{a}^{z_{i}}$ is the activation parameter of the dynamical process *z*_*i*_ and $k_{d}^{z_{i}}$ is the deactivation parameter of *z*_*i*_. To characterize the dynamical signaling features of the activated kinase signal, a transfer function model can be derived by taking the Laplace transformation on each of these differential equations. Considering all three processes as analogous to each other in their behavior yields the following form.

$$\begin{array}{c} L\left\{\dot{z}\left(t\right)\right\}=L\left\{k_{a}^{z}u\left(t\right)\right\}-L\left\{k_{d}^{z}z\left(t\right)\right\} \end{array}$$ (6)

$$\begin{array}{c} s\hat{z}\left(s\right)=k_{a}^{z}\hat{u}\left(s\right)-k_{d}^{z}\hat{z}\left(s\right) \end{array}$$ (7)

$$\begin{array}{c} F\left(s\right)=\hat{z}\left(s\right)=(\frac{k_{a}^{z}/k_{d}^{z}}{(1/k_{d}^{z})s+1})\hat{u}\left(s\right)=(\frac{\lambda }{\tau s+1})\hat{u}\left(s\right) \end{array}$$ (8)

For *u*(*t*) as a heavyside unit step function, the inverse laplace transformation (${ℒ}^{-1}\{F(s)\}$) results in an exponential ascent or decay model depending on the process [81]. Note that each of the activation, peak, and deactivation dynamical processes are defined with specified delays, *α*_*i*_, with a following constraint; *α*_1_ + *α*_2_ + *α*_3_ ≤ (*t*_*f*_ − *t*_*i*_), where *α*_1_ is the delay in activation processes, *α*_1_ + *α*_2_ is the delay in peak processes, *α*_1_ + *α*_2_ + *α*_3_ is the delay in deactivation processes, *t*_*i*_ is the initial time point and *t*_*f*_ is the final time point. Resulting dynamics of the signaling kinase typically follow a transient signaling pattern over minute timescales [79, 82]. This transient signaling profile, represented by *f*_*s*_ in Eq (9), was observed to have four phases of dynamics: delay, activation, peak retention and deactivation phases. The mathematical form for last three phases are represented as a function of two independent features: (1) a duration feature, *α* and (2) a time constant feature, *τ*, as shown in Eq (9). The resulting function of signaling dynamics is continuous and differentiable within a given boundary condition (from *t*_*i*_ = 0 to *t*_*f*_ = 60 minutes in our case) (See Fig 2A for a graphical representation).

$$\begin{array}{c} f_{s}\left(X,t\right)=\lambda \{\begin{array}{ccc} 0, & t\leq \alpha_{1} & \text{Delay}\\ 1-\exp(-\frac{\left(t-\alpha_{1}\right)}{\tau_{1}}), & \alpha_{1}<t\leq \left(\alpha_{1}+\alpha_{2}\right) & \text{Activation}\\ \exp(-\frac{\left(t-\alpha_{1}-\alpha_{2}\right)}{\tau_{2}}), & \left(\alpha_{1}+\alpha_{2}\right)<t\leq \left(\alpha_{1}+\alpha_{2}+\alpha_{3}\right) & \text{Peak Retention}\\ \exp(-\frac{\left(t-\alpha_{1}-\alpha_{2}-\alpha_{3}\right)}{\tau_{3}}), & (\alpha_{1}+\alpha_{2}+\alpha_{3})<t\leq 60 & \text{Deactivation} \end{array} \end{array}$$ (9)

where **X** ∈ {*α*_1_, *τ*_1_, *α*_2_, *τ*_2_, *α*_3_, *τ*_3_} represents the collective set of all signaling features of a kinase signal. The signaling dynamics of each activated kinase is represented by six independent features, making it a total of eighteen for three kinases ERK, FRK and JNK, respectively. List of all independent features, their least square fitted values with their appropriate experimental observations, and the range of induced variation to each signaling feature are reported in Table 1. To computationally depict the variability of single cells, duration features which are in time domain were varied up to a multiplicative factor of 2 to 4, whereas time constant features were varied up to two-fold.

### Variance based global sensitivity analysis

In this study, a biochemical regulatory network is considered as the channel which processes various dynamical aspects of the signaling pattern and generates a downstream response pattern (Fig 1). Because multiple interactions occur simultaneously in a signaling pathway, a true mathematical relationship between the dynamical signaling features and a downstream response is difficult to construct. Hence, to evaluate the influence of signaling features on a downstream response, we turn to global sensitivity analysis. Global sensitivity analysis investigates how uncertainty in key signaling features of a biochemical model affects uncertainty in its response and quantify the relative importance of these features. We here use Sobol’s high dimensional model representation (HDMR) of biochemical regulatory networks to perform variance based global sensitivity analysis and to quantify the influence of signaling features [42, 83]. The gene regulatory model is considered as a multivariate function of signaling features **X** ∈ {*α*_1_, *τ*_1_, …, *α*_*n*_, *τ*_*n*_}, where these features acts as inputs, influencing the output IEG or TF responses, ℱ, either in an independent and/or cooperative way as shown below.

$$\begin{array}{c} F\left(X\right)=f_{0}+\sum_{i\leq 2n}f_{i}\left(X_{i}\right)+\sum_{1\leq i<j\leq 2n}f_{ij}\left(X_{i},X_{j}\right)+\;\cdots \;+f_{1,2,\cdots ,2n}\left(X_{i},X_{2},\cdots ,X_{2n}\right) \end{array}$$ (10)

where $ℱ(\text{X})\equiv ℱ\left(t,{\text{y}}_{0},\text{k},f_{s}(\text{X},t)\right)$ as shown in Eq (1), *f*_0_ denotes the mean value of the ℱ(X) over the entire variation range of **X**. The first order function *f*_*i*_(*X*_*i*_) describes the independent behavior of *X*_*i*_ on output ℱ(X). In addition, the functions also preserve a nonlinearity which is essential for establishing a general case. The second order function *f*_*ij*_(*X*_*i*_, *X*_*j*_) represents the cooperative behavior between two input features *X*_*i*_ and *X*_*j*_ on output ℱ(X). Similarly, 2*n* ^th^ order functions represents the cooperative effects of 2*n* signaling features acting together to influence the output ℱ(X).

We began this analysis by considering variability in signaling dynamics as a function of variability in signaling features, *α*_*i*_ and *τ*_*i*_ in Eq (9), as a result of continuous intrinsic and extrinsic fluctuations in cells [25]. The simultaneous Monte-Carlo sampling for each signaling feature was employed to induce variability, in consistent with experimental observation, using function ‘sobolset’ and ‘scramble’ in the Matlab computational environment [84] (Table 1 for details on range of variation). HDMR decomposition measures the individual as well as higher order cooperative measurements of the variations in output (downstream response) in terms of variations in input(s) (or signaling feature(s)).

$$\begin{array}{c} F\left(X\right)\approx \underset{\text{first}\;\text{order}}{\underset{︸}{\sum_{i\leq 2n}f_{i}\left(X_{i}\right)}}+\underset{\text{higher}\;\text{order}}{\underset{︸}{\left(\sum_{1\leq i<j\leq 2n}f_{ij}\left(X_{i},X_{j}\right)+\;\cdots \;+f_{1,2,\cdots ,2n}\left(X_{i},X_{2},\cdots ,X_{2n}\right)\right)}} \end{array}$$ (11)

Total variance *V*[*R*] for a response output R≡ℱ(X) can be decomposed into individual variances of each HDMR component function using mutual orthogonality [83, 85]. The response *R* is the activity level of the downstream IEG or TF under observation. This is a very useful property of HDMR in determining how the uncertainty in downstream response (*R*) is influenced by the uncertainties in signaling features (**X**).

$$\begin{array}{c} V\left[R\right]=\sum_{i\leq 2n}V\left(E\left[R|X_{i}\right]\right)+\sum_{1\leq i<j\leq 2n}V\left(E\left[R|X_{i},X_{j}\right]\right)+\;\cdots \;+V\left(E\left[R|X_{1},X_{2},\cdots ,X_{2n}\right]\right) \end{array}$$ (12)

When variation in each feature is assumed to be unbiased (i.e. *X*_*i*_’s having a uniform distribution) S1 Fig, the Sobol sensitivity indices are defined as the ratio of decomposed partial variances to the total variance as shown below, where *S*_*i*_ represents first order sensitivity indices, and $S_{i}^{T}$ represents total sensitivity indices.

$$\begin{array}{c} S_{i}=\frac{V(E\left[R|X_{i}\right])}{V[R]} \end{array}$$ (13)

$$\begin{array}{c} S_{i}^{T}=\frac{V\left(E\left[R|X_{i}\right]\right)+\sum_{ij}V\left(E\left[R|X_{i},X_{j}\right]\right)+\;\cdots \;+V\left(E\left[R|X_{1},X_{2},\cdots ,X_{2n}\right]\right)}{V[R]} \end{array}$$ (14)

The difference between the two $S_{i}^{T}-S_{i}$ is considered as the higher order sensitivity index.

The conditional variance in the sensitivity indices were estimated using Saltelli’s approach [86]. While estimating the indices, we assume that the biochemical system responded sufficiently and quickly to reach steady state before there was any change in the signaling feature (otherwise the system would still be responding to a previous fluctuation in the features when the next change happened).

The objective of the global sensitivity analysis is to identify the most important signaling feature affecting a downstream response at a given time. This is determined through identification of a feature which, when varied simultaneously along with all the features, leads to the greatest reduction in the variance of the downstream response. Similarly, second most important feature can be determined which leads to second greatest reduction in the variance of the downstream response, and so on until all features are ranked in the order of importance. Estimation of sensitivity indices captures this reduction in the variance through a continuous metric, bounded between 0 and 1 [86]. A sensitivity index of zero means that the associated feature is non-influential to a downstream response, while the higher the index the more influential the feature. If the sensitivity indices were lower than a preestablished threshold value (in our case *S*_*i*_ < 0.1), we assumed that the signaling feature was not sensitive enough to lead to significant reduction in the response variation and was not important. This criteria was used throughout the study and in generation of the heatmap in Fig 8.

### Estimating feature-based mutual information

We applied information theory to quantify the amount of information transduced by each upstream signaling feature to downstream responses [87]. Feature-based mutual information is a quantity that measures the amount of information which can be predicted about a response, *R*, when the measurement of a signaling feature, *X*_*i*_, is known. The quantity is calculated for each feature to any downstream response at a given time point. Feature based mutual information is a universal quantity by definition, and can be applied to any network topology regardless of its underlying physical basis or complexity. In biological networks, the network topology can be defined as a channel (Fig 1A) or a black box that maps the input functions of signaling dynamics to a downstream response. Because the distribution of the input signals are typically not known *a priori*, they can be considered as a function of random variables (signaling features) given as *X* in Eq (9).

For a input signal *S* = *f*_*s*_(**X**, *t*), and downstream response *R*, mutual information *I* is defined as:

$$\begin{array}{c} I\left(R;X_{i}\right)\equiv H\left(R\right)-H\left(R|X_{i}\right)=H\left(X_{i}\right)-H\left(X_{i}|R\right)=I\left(X_{i};R\right) \end{array}$$ (15)

where entropy *H* is

$$\begin{array}{c} H\left(R\right)\equiv E_{p}\left\{-\log\left(p\left(R\right)\right)\right\}=-\sum_{R}p_{R}\left(r\right)\log\left(p_{R}\left(r\right)\right) \end{array}$$

and conditional entropy

$$\begin{array}{ccc} H(R|X_{i}) & = & E_{p}\left\{-\log\left(p\left(R|X_{i}\right)\right)\right\} \end{array}$$ (16)

$$\begin{array}{ccc} & = & -\sum_{X_{i}}p_{X_{i}}\left(x_{i}\right)\sum_{R}p_{R|X_{i}}\left(r|x_{i}\right)\log\left(p_{R|X_{i}}\left(r|x_{i}\right)\right) \end{array}$$ (17)

$$\begin{array}{ccc} & = & -\sum_{X_{i}}p_{X_{i}}\left(x_{i}\right)H\left(R|X_{i}=x_{i}\right) \end{array}$$ (18)

Since mutual information is always positive (*I*(*X*_*i*_;*R*) ≥ 0), the conditional entropy is always less than individual entropy, *H*(*R*∣*X*_*i*_) ≤ *H*(*R*) [87]. The resulting mutual information *I*(*X*_*i*_;*R*) is then a concave function of *p*_*R*_(*r*) for fixed *p*_*R*∣*X*_*i*__(*r*∣*x*_*i*_) and a convex function of *p*_*R*∣*X*_*i*__(*r*∣*x*_*i*_) for fixed *p*_*R*_(*r*). The conditional distribution characterizes these measurements.

$$\begin{array}{ccc} I(X_{i};R) & = & E\{\log(\frac{p_{X_{i}R}\left(X_{i},R\right)}{p_{X_{i}}\left(X_{i}\right)p_{R}\left(R\right)})\} \end{array}$$ (19)

$$\begin{array}{ccc} & = & \sum_{R}\sum_{X_{i}}P_{R|X_{i}}\left(r|x_{i}\right)P_{X_{i}}\left(x_{i}\right)\log(\frac{P_{R|X_{i}}\left(r|x_{i}\right)}{\sum P_{R|X_{i}}\left(r|x_{i}\right)p_{X_{i}}\left(x_{i}\right)}) \end{array}$$ (20)

$$\begin{array}{ccc} & = & \sum_{X_{i}}P_{X_{i}}\left(x_{i}\right)(H\left(p_{X_{i}}\left(x_{i}\right)\right)-H\left(P\left(R|X_{i}\right)\right)) \end{array}$$ (21)

The uniform distribution of signaling features *X*_*i*_ ensures the maximum possible entropy of $H(P_{X_{i}}(x_{i}))=\log\left(\frac{1}{max(X_{i})-min(X_{i})}\right)$. The intuition that the conditional distribution *P*(*R*∣*X*_*i*_) is narrower or more concentrated than *P*_*X*_*i*__(*x*_*i*_) is quantified by the fact that the entropy *H*(*P*(*R*∣*X*_*i*_)) is smaller than *H*(*P*_*X*_*i*__(*x*_*i*_)), and this reduction in entropy is exactly the information when observing *R* provides about *X*_*i*_, measured here in bits [88]. However, it should be noted that this equation allows us to measure the information carried by the response to recover estimates of the signaling features in each cells. We then assumed that the total as well as conditional distribution of downstream responses emerging from signaling activation followed a Gaussian distribution, whose variance was not dependent on *X*_*i*_. In mathematical terms, this means *V*[*R*∣*X*_*i*_] = *E*_*X*_*i*__(*V*[*R*∣*X*_*i*_]). From the law of total variance *V*[*R*] = *V*(*E*[*R*∣*X*_*i*_]) + *E*(*V*[*R*∣*X*_*i*_]), mutual information is estimated as:

$$\begin{array}{ccc} I(R;X_{i}) & = & H\left(R\right)-H\left(R|X_{i}\right)=\frac{1}{2}\log\left(2\pi eV\left[R\right]\right)-\frac{1}{2}\log(2\pi eV[R|X_{i}]) \end{array}$$ (22)

$$\begin{array}{ccc} & = & -\frac{1}{2}\log\frac{E(V\left[R|X_{i}\right])}{V[R]}=-\frac{1}{2}\log\frac{V\left[R\right]-V(E\left[R|X_{i}\right])}{V[R]} \end{array}$$ (23)

$$\begin{array}{ccc} & = & -\frac{1}{2}\log(1-\frac{V(E\left[R|X_{i}\right])}{V[R]}) \end{array}$$ (24)

where entropy (*H*) of any Gaussian distribution *p*_*Z*_(*z*), is $H(Z)\equiv \frac{1}{2}\log(2\pi eV[Z])$ [60, 87]. The information content between *R* and *X*_*i*_ in Eq (24) was estimated using Eq (13). Since both the signaling feature and and the output response measure are assumed to be a Gaussian, the estimation of the mutual information gives us a lower bound on the information carried by a signaling feature to the downstream response [89]. We later make a generalization that the information is not conveyed by just one single signaling feature, but by a combination of features, as it was the case revealed during the analysis. It is also important to emphasize that the number of bits of information carried by a single feature has a meaning irrespective of the dynamic multiplexing resulting from the network topology.

### Decision tree analysis

We employed decision tree analysis by generating 10^5^ sets of simultaneous variation in the signaling features through application of Sobol sequence ‘MatousekAffineOwen’ algorithm to scramble a total of 18-dimensional pseudo-random numbers generated using Sobol’s method in MATLAB computational environment [84]. The responses to these variations were statistically grouped into three categories: High, Mid and Low phenotypes. High phenotype represented most positive deviation from the mean of the downstream response at the time point under consideration, Low phenotype meant the most negative deviation from the mean of the downstream response, and Mid represented the median of the response distribution. We then selected first 250 profiles from these groups to identify the rules of the binary tree, using the classification and regression trees (CART) algorithm [43]. The CART algorithm uncovers the predictive structure of how cells might be classified in various phenotypic groups based on variations in dynamical features of the upstream signals. The objective is to progressively split the cells (or predictor dataset) into smaller and smaller subsets until each subset corresponds to only one phenotype (or a leaf of the tree). Each subset represent a hierarchy of binary rules defining the ranges of variation in the signaling features determined through a splitting criteria. In our study, the Gini index was used as the splitting criteria to construct the trees. The analysis was performed in the R statistical language [90] using the ‘rpart’ and the‘rpart.plot’ packages [91, 92].

## Supporting Information

S1 Table Details of feed-forward network motif for ERK/FRK module. The citation in the table refers to [40].
(PDF)

S2 Table Details of positive feedback loop motif for JNK module. The citation in the table refers to [40].
(PDF)

S1 Fig Control block diagram of the phenomenological model of dynamics of signaling kinase and pairwise 2d distribution between signaling features. (A) Control block diagram of signaling kinase dynamics, modeled as an outcome of enacting activation, peak and deactivation processes in parallel. Symbols used in the diagram are the same as in the Methods section of the main text. *u*(*t*) represents a input stimulus, which is assumed to be a heavyside step function, *f*_*s*_(*t*) is the resulting transient signal of the phosphorylated (activated) kinase, *z*_1_(*t*), *z*_2_(*t*) and *z*_3_(*t*) are the activation, peak and deactivation processes respectively, and *α*_*i*_ are the delays (See Fig 2A for graphical representation). (B) Pairwise 2D histogram between randomly generated features using the Sobol sequence method (See Methods section for details). Left half triangle of the heatmap shows the 2d smooth scatter plot between all 18 features. It is important to note that the Pearson correlation coefficient between between features was zero.
(PDF)

S2 Fig Modular representation of the gene regulatory network. AP-1 regulatory network has two distinct motifs: feedforward motif activating heterodimer ppc-FOS:ppc-JUN (left) and positive feedback loop activating homodimer ppc-JUN:ppc-JUN (right).
(PDF)

S3 FigDecomposing downstream transcription factor variability and measuring higher order sensitivity indices.(A) Plots show total sensitivity ($S_{i}^{T}$) indices to ppc-FOS:ppc-JUN, ppc-JUN:ppc-JUN, and Total AP-1 TF. The color bar on the top of each plot represents the feature with maximum $S_{i}^{T}$’s at that particular time. The values are shown between 5 and 55 minutes. Refer Fig 3 for details. (B) Higher order sensitivity indices ($S_{i}^{T}-S_{i}$) to ppc-FOS:ppc-JUN, ppc-JUN:ppc-JUN, and Total AP-1 TF.(PDF)

S4 FigFirst order as well as higher order sensitivity indices to additional dynamic decoders of the regulatory network.Density plot of the 100,000 simulation profiles, first order sensitivity indices (*S*_*i*_) and higher order sensitivity indices ($S_{i}^{T}-S_{i}$) to ppATF2, ppc-JUN, pp-ATF2:ppc-JUN, p-ELK1 and ppc-FOS.(PDF)

S5 Fig Initial delay and duration of the activation of signaling dynamics stratified the early response transcription factors phenotypes. Plots similar to Fig 5 but for early time point (20 min).
(PDF)

S6 Fig Activation and deactivation kinetics of signaling dynamics stratified the intermediate response transcription factor phenotypes. Plots similar to Fig 5 but for intermediate time point (40 min).
(PDF)
