# Supplementary material for: Intracellular Information Processing through Encoding and Decoding of Dynamic Signaling Features
Source: PLoS Comput Biol. 2015 Oct 22;11(10):e1004563. doi: 10.1371/journal.pcbi.1004563 (PMC4619640; doi:10.1371/journal.pcbi.1004563)
Supplement: S1 Table — The citation in the table refers to [40]. (PDF) [file pcbi.1004563.s001.pdf]

S1 Table: **Details of feed-forward network motif for ERK/FRK module.**

Species	*Equation*	*Ref.*
ELK1	$\dot{X}_1 = V_{z_1}^{x_1} \cdot f(Z_1, K_{z_1}^{x_1}) - k_{x_1}^{z_1} \cdot f(X_1, K_{x_1}^{z_1}) \cdot f_s^{\mathrm{ERK}}$	
p-ELK1	$\dot{Z}_1 = k_{x_1}^{z_1} \cdot f(X_1, K_{x_1}^{z_1}) \cdot f_s^{\mathrm{ERK}} - V_{z_1}^{x_1} \cdot f(Z_1, K_{z_1}^{x_1}) - k_{z_1}^{c} X_2^{pr} Z_1 + k_c^{z_1} C$	
c-Fos_{pr}:p-ELK1	$\dot{C} = k_{z_1}^{c} X_2^{pr} Z_1 - k_c^{z_1} C$	
$\text{c-Fos}_{\text{pre-mRNA}}$	$\dot{X}_2^{pre_{mRNA}} = R_0 + k_c^{pre_{mRNA}} C - \frac{V_c}{V_n} \cdot k_{pre_{mRNA}}^{mRNA} X_2^{pre_{mRNA}}$	
$\text{c-Fos}_{\text{mRNA}}$	$\dot{X}_2^{mRNA} = \frac{V_n}{V_c} \cdot k_{pre_{mRNA}}^{mRNA} X_2^{pre_{mRNA}} - k_{mRNA}^{\phi} X_2^{mRNA}$	
c-FOS_{p}	$\dot{X}_2^{p} = k_{mRNA}^{p} X_2^{mRNA} - k_{cyt}^{n} X_2^{p}$	[1]
c-FOS	$\dot{X}_2 = \frac{V_c}{V_n} \cdot k_{cyt}^{n} X_2^{p} + V_{y_2}^{x_2} \cdot f(Y_2, K_{y_2}^{x_2}) + V_D^{x_2,x_2^*} \cdot f(D, K_D^{x_2,x_2^*}) - k_{x_2}^{y_2} \cdot f(X_2, K_{x_2}^{y_2}) \cdot f_s^{\mathrm{FRK}} - k_{x_2}^{\phi} X_2$	
pc-FOS	$\dot{Y}_2 = k_{x_2}^{y_2} \cdot f(X_2, K_{x_2}^{y_2}) \cdot f_s^{\mathrm{FRK}} + V_{z_2}^{y_2} \cdot f(Z_2, K_{z_2}^{y_2}) - k_{y_2}^{z_2} \cdot f(Z_2, K_{y_2}^{z_2}) \cdot f_s^{\mathrm{FRK}} - V_{y_2}^{x_2} \cdot f(Y_2, K_{y_2}^{x_2}) - k_{y_2}^{\phi} Y_2$	
ppc-FOS	$\dot{Z}_2 = k_{y_2}^{z_2} \cdot f(Z_2, K_{y_2}^{z_2}) \cdot f_s^{\mathrm{FRK}} - V_{z_2}^{y_2} \cdot f(Z_2, K_{z_2}^{y_2}) - k_{z_2,z_2^*}^{D} Z_2 Z_2^* + k_D^{z_2,z_2^*} D - k_{z_2}^{\phi} Z_2$	
ppc-FOS:ppc-JUN	$\dot{D} = k_{z_2,z_2^*}^{D} Z_2 Z_2^* - k_D^{z_2,z_2^*} D - V_D^{x_2,x_2^*} \cdot f(D, K_D^{x_2,x_2^*}) - k_D^{\phi} D$	

$$V_{z_1}^{x_1} = 4.5337 \text{ nM s}^{-1}, \quad K_{z_1}^{x_1} = 9.5340 \text{ nM}, \quad k_{x_1}^{z_1} = 0.2705 \text{ s}^{-1}, \quad K_{x_1}^{z_1} = 65.2625 \text{ nM}, \quad k_{z_1}^{c} = 3.4048 \text{ nM s}^{-1}$$

$$k_c^{z_1} = 3.1303 \text{ nM}, \quad R_0 = 8.2285 \times 10^{-5} \text{ nM s}^{-1}, \quad k_c^{pre_{mRNA}} = 0.4274 \text{ s}^{-1}, \quad k_{pre_{mRNA}}^{mRNA} = 0.3316 \text{ s}^{-1}$$

$$k_{mRNA}^{\phi} = 0.1557 \text{ s}^{-1}, \quad k_{mRNA}^{p} = 16.6871 \text{ s}^{-1}, \quad k_{cyt}^{n} = 2.6297 \text{ s}^{-1}, \quad V_{y_2}^{x_2} = 8.4053 \text{ nM s}^{-1}, \quad K_{y_2}^{x_2} = 6.8804 \text{ nM}$$

$$V_D^{x_2,x_2^*} = 55.9578 \text{ nM s}^{-1}, \quad K_D^{x_2,x_2^*} = 8.947 \text{ nM}, \quad k_{x_2}^{y_2} = 0.154 \text{ s}^{-1}, \quad K_{x_2}^{y_2} = 1.6189 \text{ nM}, \quad k_{x_2}^{\phi} = 0.2741 \text{ s}^{-1}$$

$$k_{y_2}^{z_2} = 5.1573 \text{ s}^{-1}, \quad K_{y_2}^{z_2} = 3.0176 \text{ nM}, \quad V_{z_2}^{y_2} = 3.8786 \text{ nM s}^{-1}, \quad K_{z_2}^{y_2} = 15.0773 \text{ nM}, \quad k_{z_2,z_2^*}^{D} = 0.2867 \text{ nM s}^{-1}$$

$$k_D^{z_2,z_2^*} = 1.2753 \text{ s}^{-1}, \quad k_{z_2}^{\phi} = 0.0730 \text{ s}^{-1}, \quad k_{y_2}^{\phi} = 1.1317 \text{ s}^{-1}, \quad k_D^{\phi} = 0.0774 \text{ s}^{-1}$$

$f(x,K) = \dfrac{x}{K+x}$, $f_s = f_s(\alpha, \tau, t)$, $Z_2^* =$ ppc-JUN , $X_2^* =$ c-JUN, $V_c =$ cell volume, $V_n =$ nucleus volume

[1] Miller GM, Ogunnaike BA, Schwaber JS, Vadigepalli R (2010) Robust dynamic balance of AP-1 transcription factors in a neuronal gene regulatory network. BMC Systems Biology 4: 171.
