# Supplementary material for: Intracellular Information Processing through Encoding and Decoding of Dynamic Signaling Features
Source: PLoS Comput Biol. 2015 Oct 22;11(10):e1004563. doi: 10.1371/journal.pcbi.1004563 (PMC4619640; doi:10.1371/journal.pcbi.1004563)
Supplement: S2 Table — The citation in the table refers to [40]. (PDF) [file pcbi.1004563.s002.pdf]

S2 Table: **Details of positive feedback loop motif for JNK module.**

Species	*Equation*	*Ref.*
ATF2	$\dot{X}_1 = V_{y_1}^{x_1} \cdot f(Y_1, K_{y_1}^{x_1}) + V_{c_1}^{x_1,x_2} \cdot f(C_1, K_{c_1}^{x_1,x_2}) - k_{x_1}^{y_1} \cdot f(X_1, K_{x_1}^{y_1}) \cdot f_s^{\mathrm{JNK}}$	
pATF2	$\dot{Y}_1 = k_{x_1}^{y_1} \cdot f(X_1, K_{x_1}^{y_1}) \cdot f_s^{\mathrm{JNK}} + V_{z_1}^{y_1} \cdot f(Z_1, K_{z_1}^{y_1}) - V_{y_1}^{x_1} \cdot f(Y_1, K_{y_1}^{x_1}) - k_{y_1}^{z_1} \cdot f(Y_1, K_{y_1}^{z_1}) \cdot f_s^{\mathrm{JNK}}$	
ppATF2	$\dot{Z}_1 = k_{y_1}^{z_1} \cdot f(Y_1, K_{y_1}^{z_1}) \cdot f_s^{\mathrm{JNK}} - V_{z_1}^{y_1} \cdot f(Z_1, K_{z_1}^{y_1}) - k_{z_1,z_2}^{c_1} Z_2 Z_1 + k_{c_1}^{z_1 z_2} C_1$	
ppATF2:ppc-JUN	$\dot{C}_1 = k_{z_1,z_2}^{c_1} Z_2 Z_1 - k_{c_1}^{z_1,z_2} C_1 - V_{c_1}^{x_1,x_2} \cdot f(C_1, K_{c_1}^{x_1,x_2}) - k_{c_1}^{c_2} X_2^{pr} C_1 + k_{c_2}^{c_1} C_2$	
c-Jun$_{\mathrm{pr}}$:ppATF2:ppc-JUN	$\dot{C}_2 = k_{c_1}^{c_2} X_2^{pr} C_1 - k_{c_2}^{c_1} C_2$	[1]
c-Jun$_{\text{pre-mRNA}}$	$\dot{X}_2^{pre_{mRNA}} = R_0 + k_{c_2}^{pre_{mRNA}} C_2 - \frac{V_c}{V_n} \cdot k_{pre_{mRNA}}^{mRNA} X_2^{pre_{mRNA}}$	
c-Jun$_{\mathrm{mRNA}}$	$\dot{X}_2^{mRNA} = \frac{V_n}{V_c} \cdot k_{pre_{mRNA}}^{mRNA} X_2^{pre_m RNA} - k_{mRNA}^{\phi} X_2^{mRNA}$	
c-JUN$_{\mathrm{p}}$	$\dot{X}_2^p = k_{mRNA}^p X_2^{mRNA} - k_{cyt}^n X_2^p$	
c-JUN	$\dot{X}_2 = \frac{V_c}{V_n} \cdot k_{cyt}^n X_2^p + V_{c_1}^{x_1 x_2} \cdot f(C_1, K_{c_1}^{x_1 x_2}) + V_D^{x_2,x_2^*} \cdot f(D, K_D^{x_2,x_2^*}) + 2 \cdot V_D^{x_2} \cdot f(D, K_D^{x_2}) + V_{y_2}^{x_2} \cdot f(Y_2, K_{y_2}^{x_2}) - k_{x_2}^{y_2} \cdot f(X_2, K_{x_2}^{y_2}) \cdot f_s^{\mathrm{JNK}} - k_{x_2}^{\phi} X_2$	
pc-JUN	$\dot{Y}_2 = k_{x_2}^{y_2} \cdot f(X_2, K_{x_2}^{y_2}) \cdot f_s^{\mathrm{JNK}} + V_{z_2}^{y_2} \cdot f(Z_2, K_{z_2}^{y_2}) - k_{y_2}^{z_2} \cdot f(Z_2, K_{y_2}^{z_2}) \cdot f_s^{\mathrm{JNK}} - V_{y_2}^{x_2} \cdot f(Y_2, K_{y_2}^{x_2}) - k_{y_2}^{\phi} Y_2$	
ppc-JUN	$\dot{Z}_2 = k_{y_2}^{z_2} \cdot f(Z_2, K_{y_2}^{z_2}) \cdot f_s^{\mathrm{JNK}} - V_{z_2}^{y_2} \cdot f(Z_2, K_{z_2}^{y_2}) - k_{z_2}^{\phi} Z_2 - k_{z_1,z_2}^{c_1} Z_2 Z_1 + k_{c_1}^{z_1,z_2} C_1 - 2 \cdot k_{z_2}^{D} Z_2 Z_2 + 2 \cdot k_D^{z_2} D - k_{z_2,z_2^*}^{D^*} Z_2 Z_2^* + k_{D^*}^{z_2,z_2^*} D^*$	
ppc-JUN:ppc-JUN	$\dot{D} = k_{z_2}^D Z_2 Z_2 - k_D^{z_2} D - V_D^{x_2} \cdot f(D, K_D^{x_2}) - k_D^{\phi} D$	

$V_{y_1}^{x_1} = 17.7672$ nM s^{-1}, $K_{y_1}^{x_1} = 1.7933$ nM, $V_{c_1}^{x_1,x_2} = 125.3660$ nM s^{-1}, $K_{c_1}^{x_1,x_2} = 56.3667$ nM, $k_{x_1}^{y_1} = 1.3369$ s^{-1}

$K_{x_1}^{y_1} = 21.5017$ nM, $V_{z_1}^{y_1} = 8.485$ nM s^{-1}, $K_{z_1}^{y_1} = 3.3207$ nM, $k_{y_1}^{z_1} = 1.3369$ s^{-1}, $K_{y_1}^{z_1} = 21.5017$ nM

$k_{z_1,z_2}^{c_1} = 0.9128$ nM s^{-1}, $k_{c_1}^{z_1 z_2} = 1.7441$ s^{-1}, $k_{c_1}^{c_2} = 0.2352$ nM s^{-1}, $k_{c_2}^{c_1} = 14.6428$ s^{-1}, $R_0 = 8.1524 \times 10^{-4}$ nM s^{-1}

$k_{c_2}^{pre_{mRNA}} = 0.4267$ s^{-1}, $k_{pre_{mRNA}}^{mRNA} = 0.6621$ s^{-1}, $k_{mRNA}^{\phi} = 0.068$ s^{-1}, $k_{mRNA}^{p} = 59.9722$ s^{-1}

$k_{cyt}^n = 2.5531$ s^{-1}, $V_{y_2}^{x_2} = 69.5154$ nM s^{-1}, $K_{y_2}^{x_2} = 112.334$ nM, $V_D^{x_2,x_2^*} = 55.9578$ nM s^{-1}, $K_D^{x_2,x_2^*} = 8.947$ nM

$k_{x_2}^{y_2} = 6.5615$ nM s^{-1}, $K_{x_2}^{y_2} = 27.1651$ nM, $k_{x_2}^{\phi} = 0.0214$ s^{-1}, $k_{y_2}^{z_2} = 1.3943$ s^{-1}, $K_{y_2}^{z_2} = 2.069$ nM

$V_{z_2}^{y_2} = 11.9303$ nM s^{-1}, $K_{z_2}^{y_2} = 110.6230$ nM, $k_{z_2,z_2^*}^{D^*} = 0.2867$ nM s^{-1}, $k_{D^*}^{z_2,z_2^*} = 1.2753$ s^{-1}, $k_{z_2}^{\phi} = 0.00090396$ s^{-1}

$k_{y_2}^{\phi} = 0.0053$ s^{-1}, $k_D^{\phi} = 0.0498$ s^{-1}, $V_D^{x_2} = 105.164$ nM s^{-1}, $K_D^{x_2} = 60.7342$ nM, $k_{z_2}^D = 0.0546$ nM s^{-1}, $k_D^{z_2} = 428.219$ s^{-1}

$f(x,K) = \dfrac{x}{K+x}$, $f_s = f_s(\alpha, \tau, t)$, $X_2^* =$ c-FOS, $Z_2^* =$ ppc-FOS, $D^* =$ ppc-FOS:ppc-JUN, $V_c =$ cell volume, $V_n =$ nucleus volume

[1] Miller GM, Ogunnaike BA, Schwaber JS, Vadigepalli R (2010) Robust dynamic balance of AP-1 transcription factors in a neuronal gene regulatory network. BMC Systems Biology 4: 171.
