# Supplementary figures and images for: Intracellular Information Processing through Encoding and Decoding of Dynamic Signaling Features
Source: PLoS Comput Biol. 2015 Oct 22;11(10):e1004563. doi: 10.1371/journal.pcbi.1004563 (PMC4619640; doi:10.1371/journal.pcbi.1004563)

A

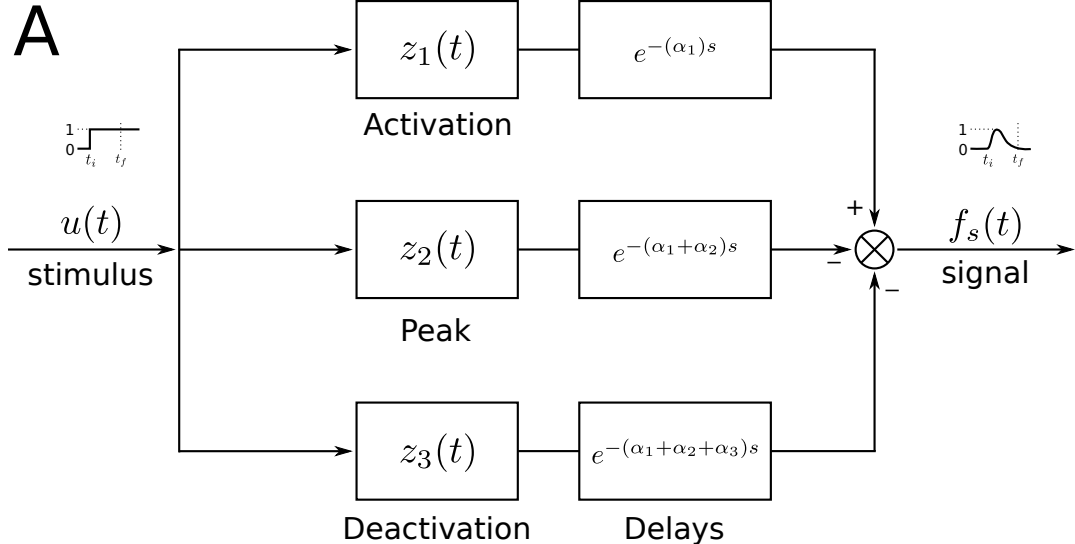

B

Pairwise Distributions between signaling features to confirm independent sampling

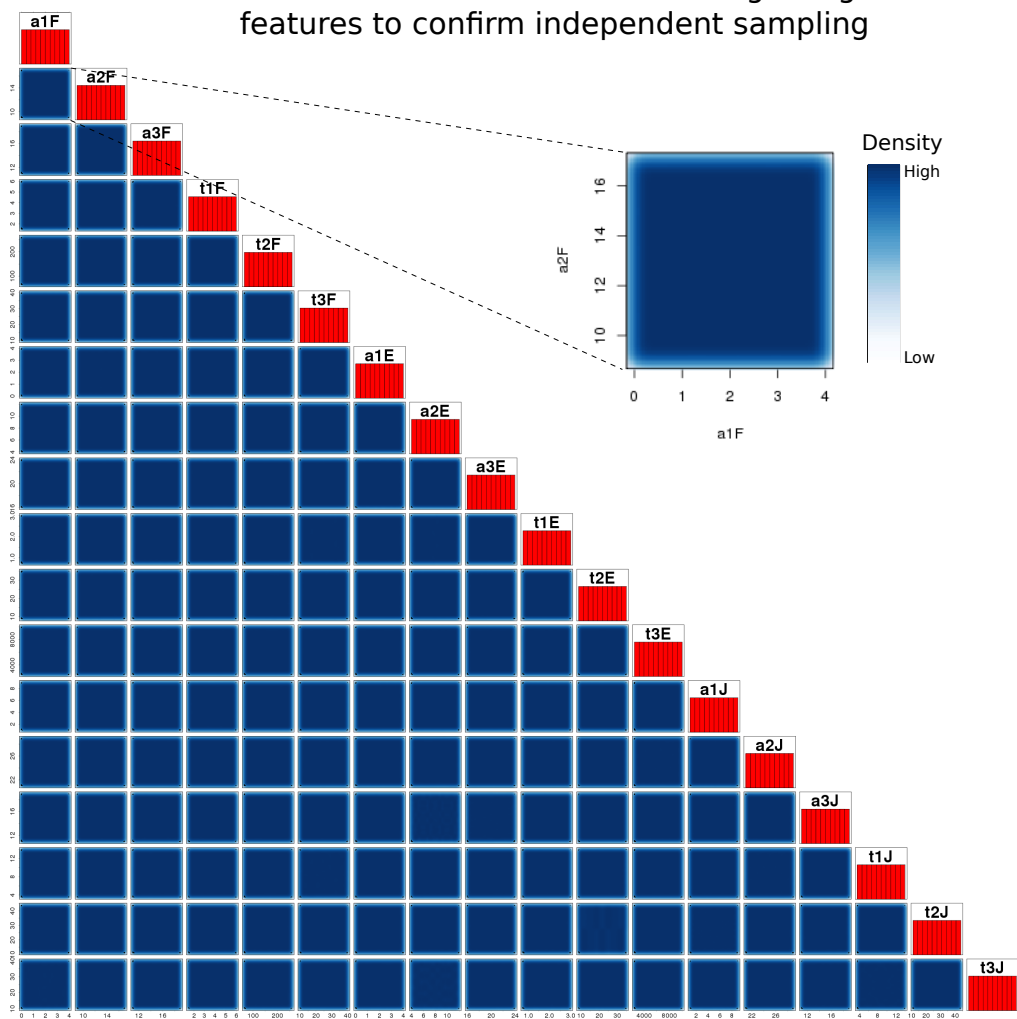

Supplement: S1 Fig — (A) Control block diagram of signaling kinase dynamics, modeled as an outcome of enacting activation, peak and deactivation processes in parallel. Symbols used in the diagram are the same as in the Methods section of the main text. u(t) represents a input stimulus, which is assumed to be a heavyside step function, f s(t) is the resulting transient signal of the phosphorylated (activated) kinase, z 1(t), z 2(t) and z 3(t) are the activation, peak and deactivation processes respectively, and α i are the delays (See Fig 2A for graphical representation). (B) Pairwise 2D histogram between randomly generated features using the Sobol sequence method (See Methods section for details). Left half triangle of the heatmap shows the 2d smooth scatter plot between all 18 features. It is important to note that the Pearson correlation coefficient between between features was zero. (PDF) [file pcbi.1004563.s003.pdf]

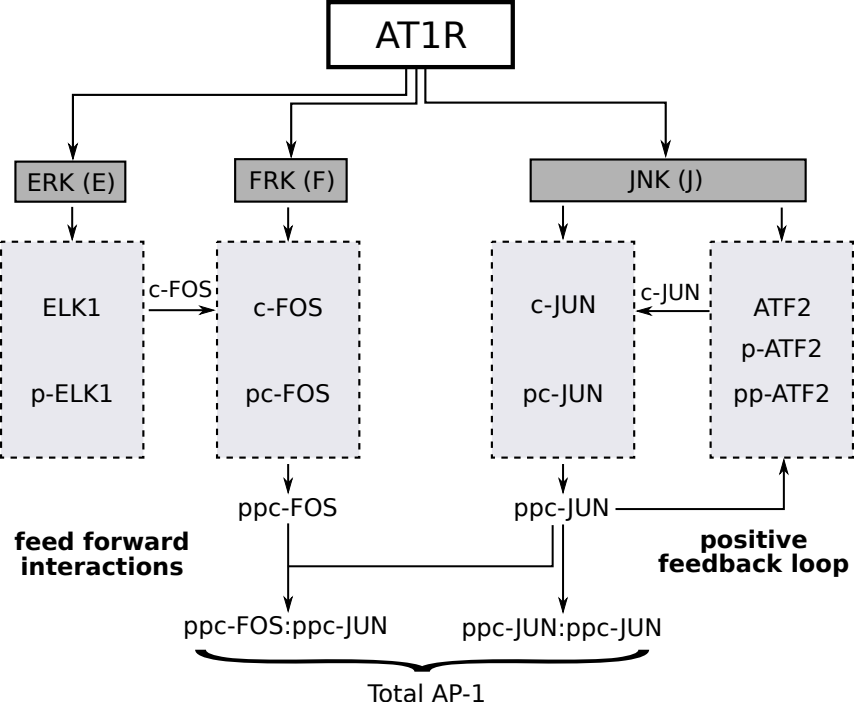

Supplement: S2 Fig — AP-1 regulatory network has two distinct motifs: feedforward motif activating heterodimer ppc-FOS:ppc-JUN (left) and positive feedback loop activating homodimer ppc-JUN:ppc-JUN (right). (PDF) [file pcbi.1004563.s004.pdf]

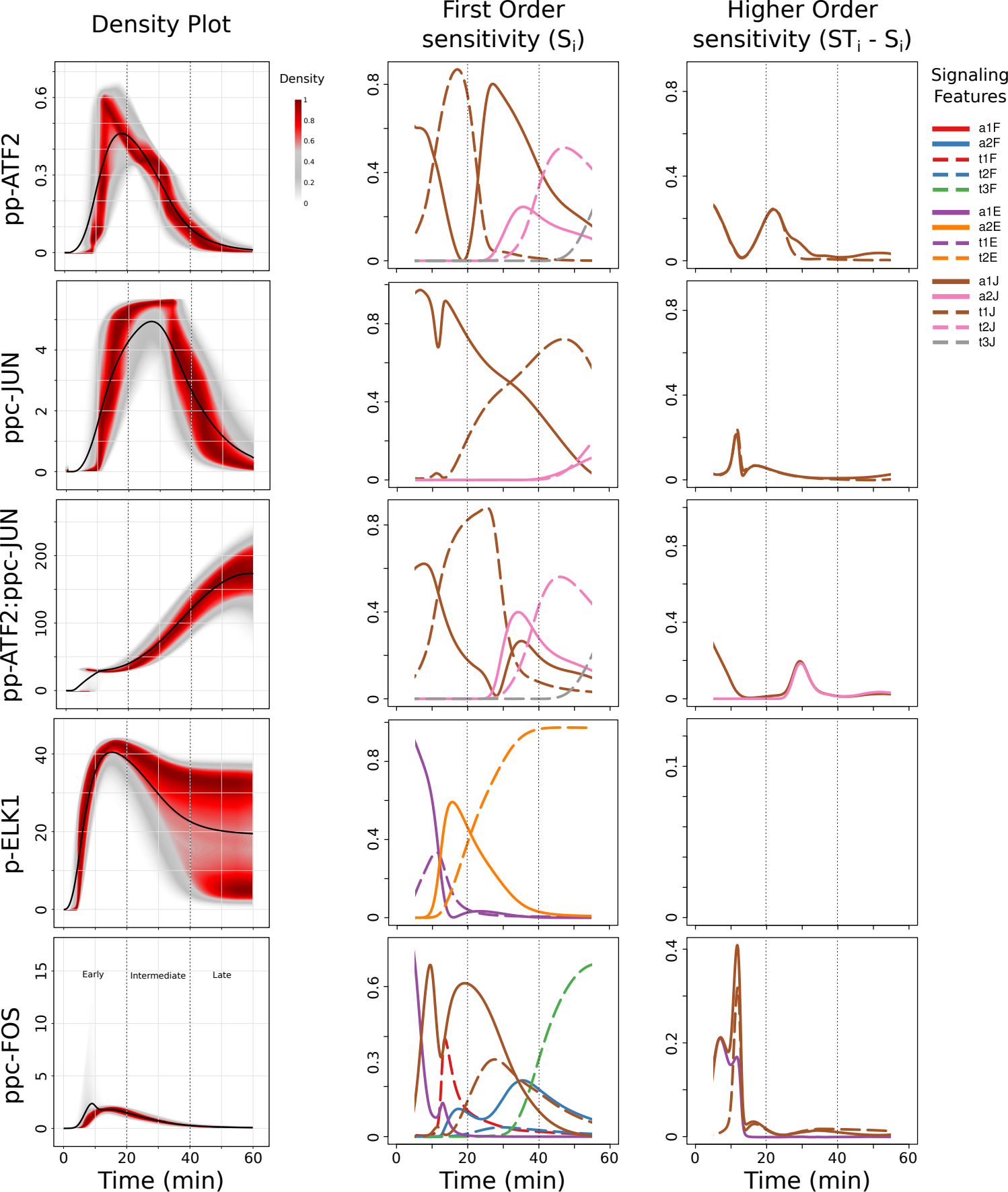

Supplement: S4 Fig — Density plot of the 100,000 simulation profiles, first order sensitivity indices (S i) and higher order sensitivity indices (SiT−Si) to ppATF2, ppc-JUN, pp-ATF2:ppc-JUN, p-ELK1 and ppc-FOS. (PDF) [file pcbi.1004563.s006.pdf]

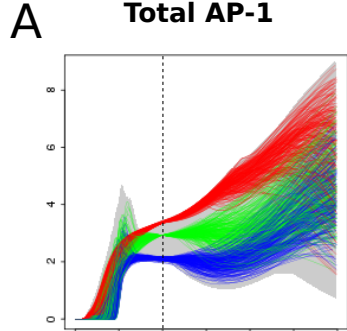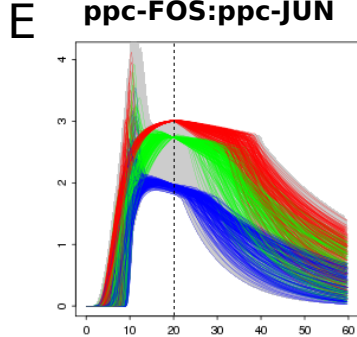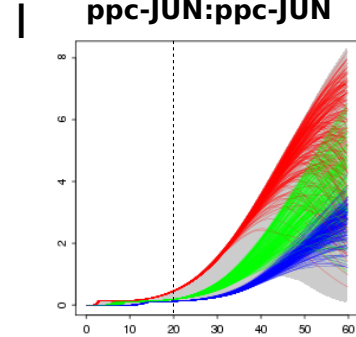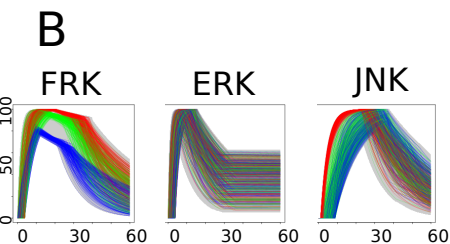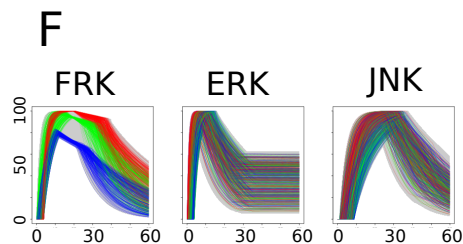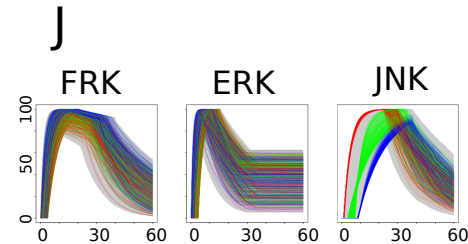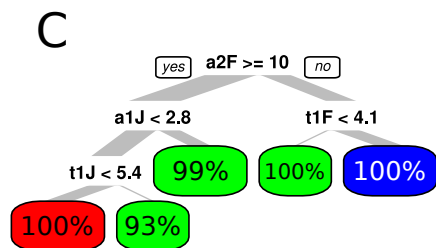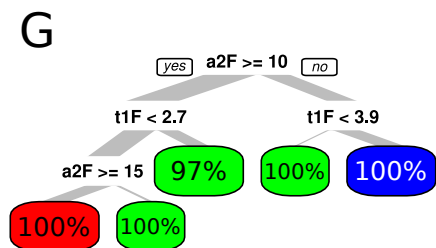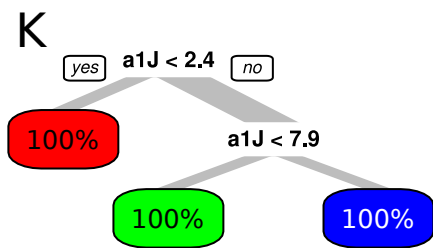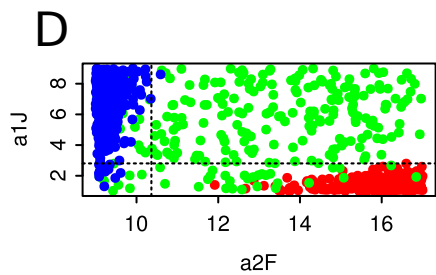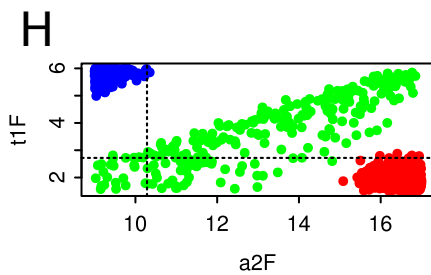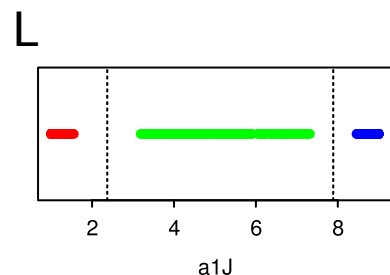

Supplement: S5 Fig — Plots similar to Fig 5 but for early time point (20 min). (PDF) [file pcbi.1004563.s007.pdf]

A

Total AP-1

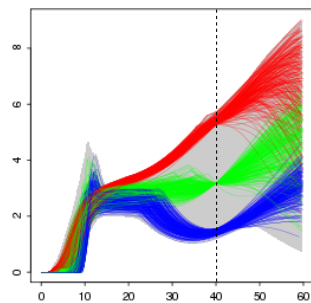

E

ppc-FOS:ppc-JUN

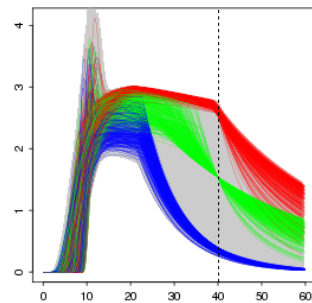

I

ppc-JUN:ppc-JUN

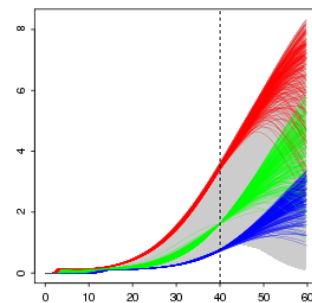

B

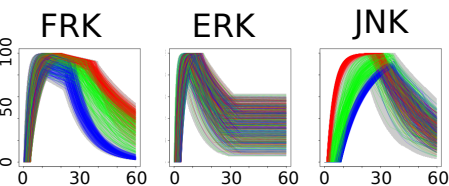

F

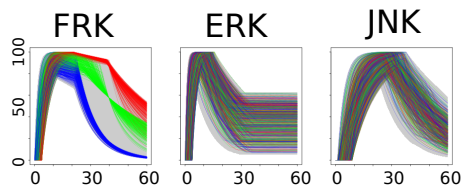

J

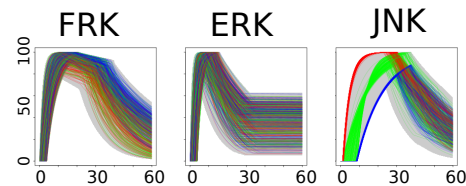

C

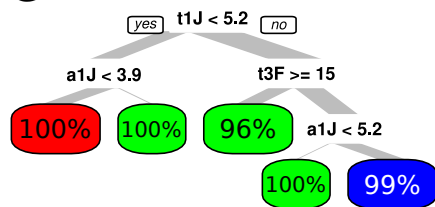

G

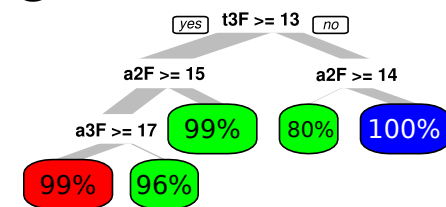

K

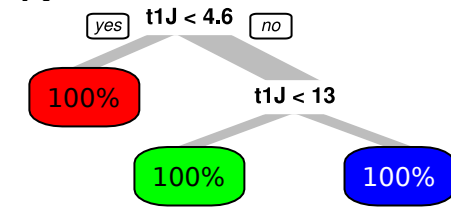

D

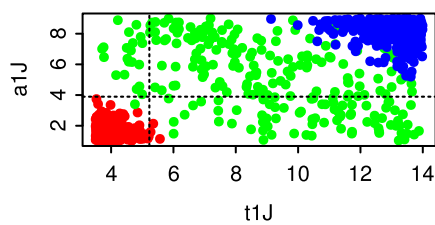

H

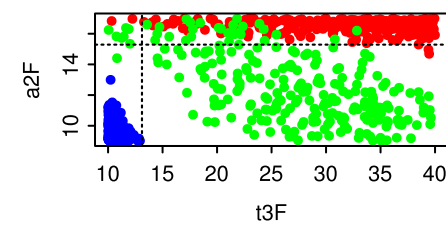

L

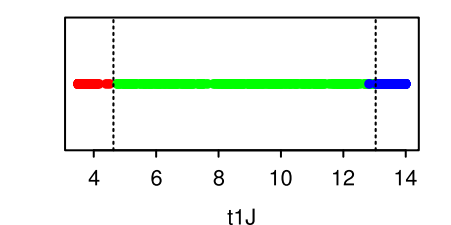

Supplement: S6 Fig — Plots similar to Fig 5 but for intermediate time point (40 min). (PDF) [file pcbi.1004563.s008.pdf]
