# Supplementary material for: Intracellular Information Processing through Encoding and Decoding of Dynamic Signaling Features
Source: PLoS Comput Biol. 2015 Oct 22;11(10):e1004563. doi: 10.1371/journal.pcbi.1004563 (PMC4619640; doi:10.1371/journal.pcbi.1004563)
Supplement: S3 Fig — (A) Plots show total sensitivity (SiT) indices to ppc-FOS:ppc-JUN, ppc-JUN:ppc-JUN, and Total AP-1 TF. The color bar on the top of each plot represents the feature with maximum SiT’s at that particular time. The values are shown between 5 and 55 minutes. Refer Fig 3 for details. (B) Higher order sensitivity indices (SiT−Si) to ppc-FOS:ppc-JUN, ppc-JUN:ppc-JUN, and Total AP-1 TF. (PDF) [file pcbi.1004563.s005.pdf]

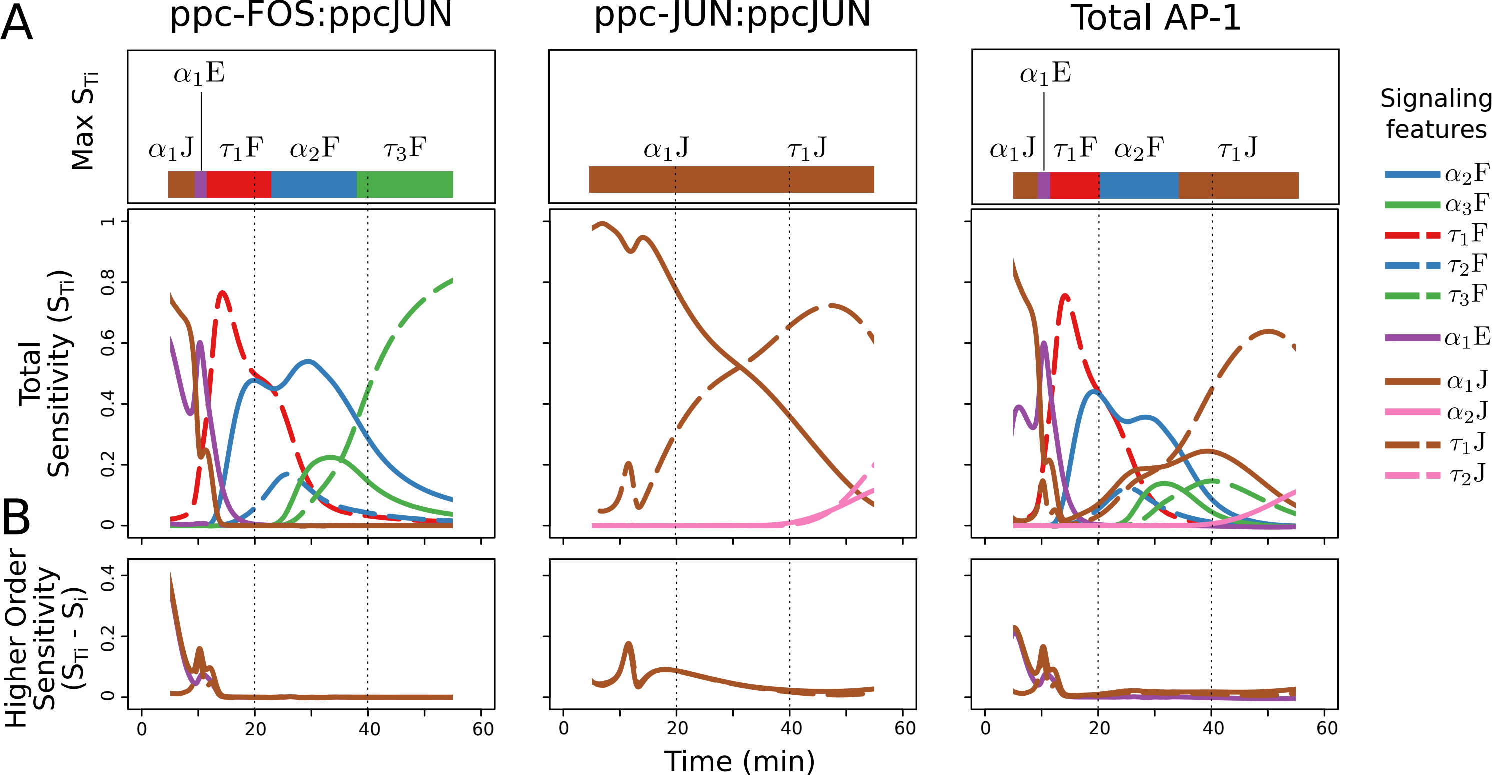

A
B
ppc-FOS:ppcJUN
ppc-JUN:ppcJUN
Total AP-1
Max S_{Ti}
α_1E
α_1J
τ_1F
α_2F
τ_3F
τ_1J
Total Sensitivity (S_{Ti})
Higher Order Sensitivity (S_{Ti} - S_i)
Time (min)
Signaling features
α_2F
α_3F
τ_1F
τ_2F
τ_3F
α_1E
α_1J
α_2J
τ_1J
τ_2J
